# Amino Acid Catabolism in *Staphylococcus aureus* and the Function of Carbon Catabolite Repression

**DOI:** 10.1128/mBio.01434-16

**Published:** 2017-02-14

**Authors:** Cortney R. Halsey, Shulei Lei, Jacqueline K. Wax, Mckenzie K. Lehman, Austin S. Nuxoll, Laurey Steinke, Marat Sadykov, Robert Powers, Paul D. Fey

**Affiliations:** a University of Nebraska Medical Center, Department of Pathology and Microbiology, Omaha, Nebraska, USA; b Department of Chemistry, University of Nebraska-Lincoln, Lincoln, Nebraska, USA; c Department of Biology, University of Nebraska at Kearney, Kearney, Nebraska, USA; d University of Nebraska Medical Center, Department of Biochemistry and Molecular Biology, Omaha, Nebraska, USA; University of Pittsburgh; Harvard Medical School

## Abstract

*Staphylococcus aureus* must rapidly adapt to a variety of carbon and nitrogen sources during invasion of a host. Within a staphylococcal abscess, preferred carbon sources such as glucose are limiting, suggesting that *S. aureus* survives through the catabolism of secondary carbon sources. *S. aureus* encodes pathways to catabolize multiple amino acids, including those that generate pyruvate, 2-oxoglutarate, and oxaloacetate. To assess amino acid catabolism, *S. aureus* JE2 and mutants were grown in complete defined medium containing 18 amino acids but lacking glucose (CDM). A mutation in the *gudB* gene, coding for glutamate dehydrogenase, which generates 2-oxoglutarate from glutamate, significantly reduced growth in CDM, suggesting that glutamate and those amino acids generating glutamate, particularly proline, serve as the major carbon source in this medium. Nuclear magnetic resonance (NMR) studies confirmed this supposition. Furthermore, a mutation in the *ackA* gene, coding for acetate kinase, also abrogated growth of JE2 in CDM, suggesting that ATP production from pyruvate-producing amino acids is also critical for growth. In addition, although a functional respiratory chain was absolutely required for growth, the oxygen consumption rate and intracellular ATP concentration were significantly lower during growth in CDM than during growth in glucose-containing media. Finally, transcriptional analyses demonstrated that expression levels of genes coding for the enzymes that synthesize glutamate from proline, arginine, and histidine are repressed by CcpA and carbon catabolite repression. These data show that pathways important for glutamate catabolism or ATP generation via Pta/AckA are important for growth in niches where glucose is not abundant, such as abscesses within skin and soft tissue infections.

## IMPORTANCE

*S. aureus* is a significant cause of both morbidity and mortality worldwide. This bacterium causes infections in a wide variety of organ systems, the most common being skin and soft tissue. Within a staphylococcal abscess, levels of glucose, a preferred carbon source, are limited due to the host immune response. Therefore, *S. aureus* must utilize other available carbon sources such as amino acids or peptides to proliferate. Our results show that glutamate and amino acids that serve as substrates for glutamate synthesis, particularly proline, function as major carbon sources during growth, whereas other amino acids that generate pyruvate are important for ATP synthesis via substrate-level phosphorylation in the Pta-AckA pathway. Our data support a model whereby certain amino acid catabolic pathways, and acquisition of those particular amino acids, are crucial for growth in niches where glucose is not abundant.

## INTRODUCTION

*Staphylococcus aureus* has the ability to establish infections in a wide variety of metabolic niches within the human host, including, among others, the heart, bone, kidney, blood, and soft tissue. Significant advances have been made in the study of bacterial virulence factors and their functions in human disease and infection of these various organ systems. However, we have only begun to study how particular carbon and nitrogen sources that are available to *S. aureus* in specific host niches affect virulence factor expression and subsequent invasion, metastasis, or quiescence. As these metabolic pathways are required for bacterial proliferation within the host, knowledge of these pathways may reveal novel avenues for drug development.

A critical component of the innate immune response is the synthesis of nitric oxide (NO) by inducible nitric oxide synthase (iNOS) within activated phagocytes (1). Nitric oxide has wide-ranging effects on bacterial cells, including the disruption of lipid biosynthesis, central metabolism, respiration, and DNA replication (2). Data from an evolving model demonstrate that, in contrast to other staphylococcal species, *S. aureus* has the ability to grow under NO stress conditions by fermenting glucose via lactate dehydrogenase, generating lactate, and facilitating NAD^+^ regeneration to maintain glycolytic flux and redox balance (3–7). Under NO stress conditions, glycolysis is an essential process, as a mutation in the glycolytic enzyme pyruvate kinase (coded for by *pyk*) severely inhibits initiation of abscess formation in a skin and soft tissue murine model (7). Indeed, inhibitors of pyruvate kinase have proved promising against *S. aureus* and other Gram-positive pathogens (8–11).

Following invasion of host tissue, *S. aureus* replicates in abscess communities that are encapsulated within fibrin deposits and infiltrating immune cells (12). Much recent work has led to an overarching view of abscess formation, including the definition of essential virulence factors such as coagulase, von Willebrand factor, ClfA, FnbpA, and FnbpB (13). As the abscess matures within the fibrin wall, the center includes a large cluster of viable cells called the staphylococcal abscess community (12, 14). It is hypothesized that these niches have low concentrations of NO, allowing respiratory activity (5, 15); however, it is predicted that *S. aureus* must grow on nonpreferred carbon sources, as glucose is not abundant (6, 16). Indeed, *S. aureus* lacks the ability to utilize phospholipids, triglycerides, and potentially other short-chain fatty acids as carbon sources due to the lack of both β-oxidation and glyoxylate shunt pathways. Therefore, other carbon sources that are available include lactate, excreted from *S. aureus* during fermentative growth, as well as peptides and free amino acids. Recent work from Spahich and colleagues showed that the addition of lactate plus peptides supports growth of *S. aureus* under conditions of low NO stress (6). Growth under these conditions was dependent upon the presence of lactate quinone oxidoreductase (Lqo; coded for by *lqo*), which assimilates lactate in an NAD-independent manner and requires an electron acceptor such as oxygen or nitrate (4). Importantly, growth under these conditions also required peptides to serve as a carbon source to facilitate gluconeogenesis.

To date, little has been known about amino acid catabolism in *S. aureus*; however, bioinformatics analysis suggests that pathways are present to catabolize multiple amino acids, including those that generate pyruvate, 2-oxoglutarate, and oxaloacetate (see Table S1 in the supplemental material). Although it is unclear in which microniches amino acid/peptide catabolism is important, it has been presumed that *S. aureus* utilizes peptides/amino acids as carbon sources as it encodes multiple oligopeptide permeases and free amino acid transporters (17–19). In addition, *S. aureus* encodes metalloenzyme, serine, and papain-like cysteine proteases that have the ability to degrade host proteins (20). We have recently understood that at least some of these amino acid catabolic pathways, such as those linking arginine and proline, are repressed by carbon catabolite repression (via CcpA) and are induced only when *S. aureus* is growing on nonpreferred carbon sources (21–23). Thus, a *S. aureus ccpA* mutant interconverts arginine and proline via an intact urea cycle (21–23).

10.1128/mBio.01434-16.6 TABLE S1  Predicted amino acid catabolism in *Staphylococcus aureus* FPR3757. Catabolic pathways were predicted using BsubCyc and EcoCyc on the MetaCyc server (metacyc.org) and UniProt. *S. aureus* orthologues were detected using the USA300_FPR3757 genome sequence (CP000255). Download TABLE S1, DOCX file, 0.1 MB. Copyright © 2017 Halsey et al. 2017 Halsey et al. This content is distributed under the terms of the Creative Commons Attribution 4.0 International license.

In this study, we utilized genetic and nuclear magnetic resonance (NMR) metabolomics approaches to investigate amino acid catabolism in *S. aureus* USA300 JE2. We found that glutamate catabolism via GudB, which functions to replenish the tricarboxylic acid (TCA) cycle via 2-oxoglutarate, is required for growth in a glucose-free medium. In addition, ATP generated from the Pta/AckA pathway is also required for growth, presumably due to a significantly reduced respiration rate. These data provide a model suggesting that certain amino acid catabolic pathways are essential for growth in niches where glucose is limiting.

## RESULTS

### Growth of *S. aureus* in complete defined medium containing 18 amino acids but lacking glucose (CDM).

**FIG 1:**
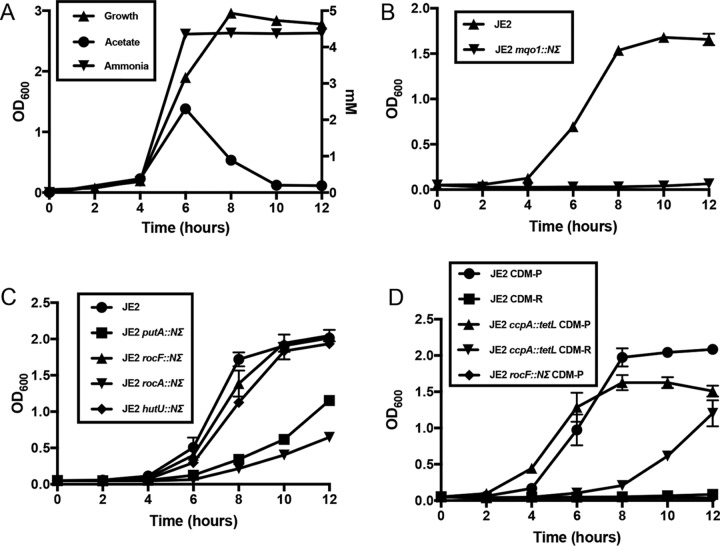
Growth of *S. aureus* JE2 in CDM. (A) Aerobic growth of JE2 in CDM displaying acetate and ammonia production. (B) Growth of JE2 and JE2 *mqo1*::*NΣ* strains in CDM lacking aspartate (CDM-D). (C) Growth of JE2 *putA*::*NΣ*, JE2 *rocF*::*NΣ*, JE2 *rocA*::*NΣ*, and JE2 *hutU*::*NΣ* strains in CDM lacking glutamate (CDM-E) in comparison to wild-type JE2. (D) Growth of JE2 *rocF*::*NΣ*, JE2 *ccpA*::*tetL*, and JE2 wild-type strains in CDM lacking proline (CDM-P) or arginine (CDM-R). Growth curve analyses whose results are shown in panels B to D were performed in three independent experiments; error bars represent the standard errors of the means (SEM). Data in panel A are representative of experiments performed three independent times.

**FIG 2:**
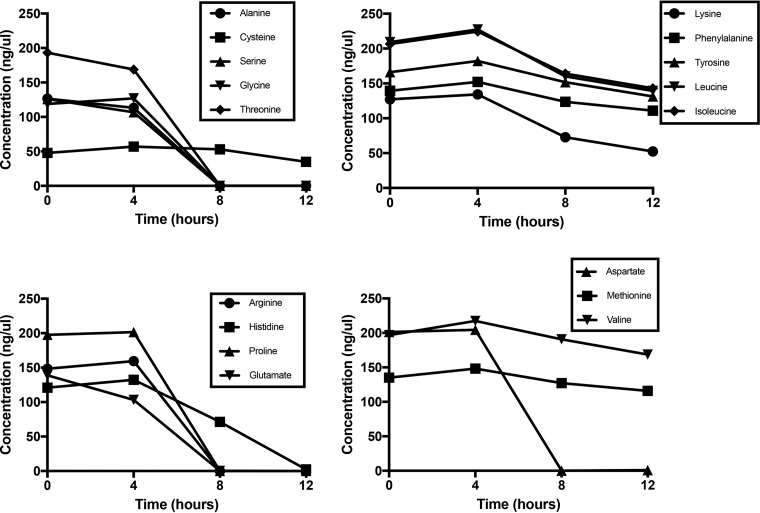
Amino acid consumption of JE2 following growth in CDM. JE2 was grown aerobically in CDM, and amino acid concentrations (millimoles) were measured in the supernatant at 0, 4, 8, and 12 h of growth. The results are representative of two independent experiments.

Staphylococcal growth and central metabolism in tryptic soy broth (TSB), which contains 14 mM glucose and an ill-defined concentration of peptides and free amino acids, have been well studied (24). Therefore, to fully assess amino acid catabolism, a complete defined medium was prepared as described by Hussain and colleagues (25) except that no glucose was added. This medium contains 18 amino acids but excludes glutamine and asparagine. Aerobic growth of JE2 in CDM reached a maximum optical density at 600 nm (OD_600_) of 3.0 following 8 h (Fig. 1A). Acetate was produced at a concentration of 2.3 mM by 6 h of growth (Fig. 1A), indicating catabolism of the glucogenic amino acids yielding pyruvate and the generation of acetate via Pta/AckA (26, 27). Acetate was subsequently consumed from the medium as a secondary carbon source and presumably oxidized via the TCA cycle (Fig. 1A) (28). Amino acid analysis of the spent medium demonstrated that alanine, serine, glycine, threonine, arginine, proline, glutamate, and aspartate were rapidly consumed by 8 h of growth, whereas histidine was not consumed until 12 h of growth (Fig. 2). Consistent with these data was the generation of ammonia, which reached a maximum concentration of 4.3 mM at 6 h of growth (Fig. 1A) and is indicative of amino acid catabolism (29). There was a gradual consumption of other amino acids (cysteine, lysine, phenylalanine, tyrosine, leucine, isoleucine, valine, and methionine) indicating probable use for protein synthesis but not for catabolic purposes (Fig. 2).

**FIG 3:**
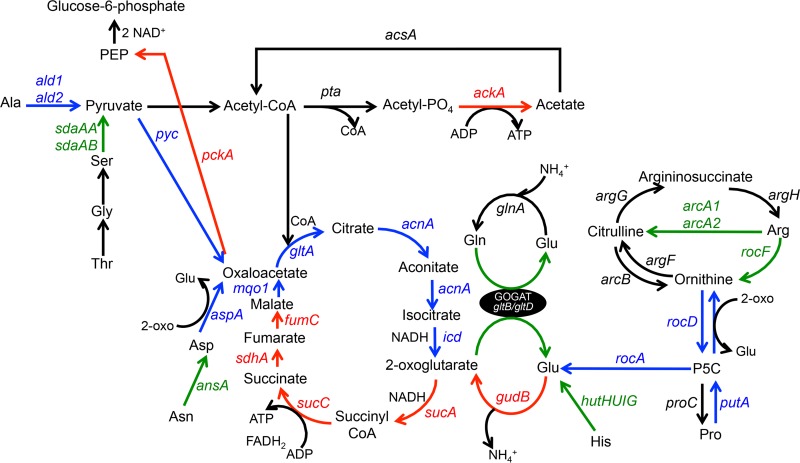
Amino acid catabolic pathways in *S. aureus*. Green arrows indicate those mutations that resulted in no significant growth defect compared to wild-type JE2 in CDM. Blue arrows indicate those mutations that resulted in intermediate growth defects compared to wild-type JE2 in CDM. Red arrows indicate mutations that completely inhibited growth of JE2 in CDM. Black arrows indicate that these pathways were not tested. Please see Fig. S1 for growth analyses.

Based on bioinformatics analyses and comparison with known pathways in *Bacillus subtilis* and *Escherichia coli*, *S. aureus* JE2 encodes genes for enzymes that function to catabolize amino acids to the metabolic intermediates pyruvate (from alanine, serine, glycine, threonine, and cysteine), 2-oxoglutarate (from glutamate, glutamine, histidine, arginine, and proline), and oxaloacetate (from aspartate and asparagine) (see Table S1 in the supplemental material). Complete catabolic pathways could not be identified for tryptophan, isoleucine, leucine, lysine, methionine, phenylalanine, tyrosine, and valine. Other than cysteine, which was not catabolized by JE2 in CDM (Fig. 2), the results of these analyses agreed with our experimental observations as detailed in Fig. 2. To determine which catabolic pathways are required for growth in CDM, mutants from each predicted pathway (see Table S1) (*ald* and *ald2*, alanine; *sdaAA*, serine, glycine, and threonine; *aspA*, aspartate; *ansa*, asparagine; *rocF*, arginine; *putA*, proline; *gudB*, glutamate; *hutU*, histidine) were chosen from a sequence-defined transposon library (30) and growth analysis was performed (Fig. 3 and S1A to I in the supplemental material). These data demonstrated that only *gudB* (see Fig. S1H), which encodes glutamate dehydrogenase and catalyzes the reaction between glutamate and 2-oxoglutarate, was required for growth in CDM. In addition, a transposon insertion within *putA* (see Fig. S1G) resulted in a growth yield defect and mutations in *ald* (see Fig. S1A) and *aspA* (see Fig. S1D) resulted in growth lag defects, although their growth rates and yields were unaffected. Note that no phenotype was detected for *ald2*, which codes for a second proposed alanine dehydrogenase (see Fig. S1B). Collectively, these data suggest that amino acids which serve to fuel pyruvate (alanine) and oxaloacetate (aspartate) synthesis are important for initiation of growth in CDM. In addition, carbon derived from glutamate and those amino acids that can be converted to glutamate, particularly proline, serves as the major carbon source for fueling the TCA cycle and subsequent gluconeogenic reactions via phosphoenolpyruvate (PEP) carboxykinase (coded for by *pckA*) (Fig. 3). On the basis of these observations, we predicted that mutations in the TCA cycle enzymes generating oxaloacetate from 2-oxoglutarate would also be required for growth. Therefore, transposon mutants in *sucA* (encoding 2-oxoglutarate dehydrogenase), *sucC* (succinyl coenzyme A [CoA] synthetase), *sdhA* (succinate dehydrogenase), *fumC* (fumarase), and *mqo1* (malate dehydrogenase) were grown in CDM (see Fig. S1J to N). As predicted, no growth was observed with the *sucA*, *sucC*, *sdhA*, and *fumC* mutants (Fig. 3; also see Fig. S1J to M). However, growth, albeit delayed, was observed with the *mqo1* mutant (Fig. 3; also see Fig. S1N). This suggests that oxaloacetate can also be supplied by aspartate/glutamate transamination via AspA. Indeed, an *mqo1* mutant was unable to grow in CDM lacking aspartate (CDM-D; Fig. 1B). However, since oxaloacetate supplied by aspartate cannot serve to complement the *sucA*, *sucC*, *sdhA*, or *fumC* mutants, these data suggest that these reactions may have critical functions other than carbon catabolism. Lastly, as predicted, no growth was observed for the *pckA* mutant, which is a critical enzyme required for gluconeogenesis via the generation of phosphoenolpyruvate from oxaloacetate (Fig. 3; also see Fig. S1O).

10.1128/mBio.01434-16.1 FIG S1  Growth of JE2 and transposon mutants in CDM. Aerobic growth (250 rpm, 10:1 flask/volume ratio, 37°C) in CDM of JE2 *bursa aurealis* mutants from the Nebraska Transposon Mutant Library. Each growth curve was assessed using three biological replicates except for the JE2 *hutU*::*NΣ* strain, for which the assessment was performed 8 separate times. Error bars represent standard errors of the means. pNF275 (panel G) and pNF311 (panel H) are the *putA* and *gudB* complementation plasmids, respectively. Download FIG S1, PDF file, 1 MB. Copyright © 2017 Halsey et al. 2017 Halsey et al. This content is distributed under the terms of the Creative Commons Attribution 4.0 International license.

The *putA* (proline), *rocF* (arginine), *hutU* (histidine), and *rocA* (arginine and proline) transposon mutants were grown in CDM lacking glutamate (CDM-E) to determine which of the amino acid substrates that fuel glutamate synthesis are most crucial (Fig. 1C). As a result, we found that the *putA* and *rocA* mutants had significant growth yield defects whereas mutations in *rocF* and *hutU* resulted in only moderate growth defects compared to wild-type JE2 grown in CDM-E. Thus, these data show that proline, arginine, and histidine all serve to fuel glutamate synthesis when glutamate is limiting; however, proline (obtained via PutA and RocA) is the major source of glutamate.

As shown in Fig. 1A, growth of JE2 in CDM produces acetate, suggesting that the glucogenic amino acids generating pyruvate (serine, threonine, glycine, and alanine) are important for ATP synthesis via substrate-level phosphorylation in the Pta/AckA pathway (27). To determine the importance of this pathway, a JE2 Δ*ackA* mutant (27) was grown in CDM and, as shown in Fig. S1P and Fig. 3, growth was significantly reduced, suggesting that generation of ATP via this pathway may be critical to growth using amino acids as carbon sources. Lastly, we found only a minor growth defect with the *pyc* mutant, suggesting that pyruvate flux is directed primarily toward acetate synthesis instead of oxaloacetate and the TCA cycle (Fig. S1Q and Fig. 3).

### NMR metabolomic analysis of amino acid catabolism in *S. aureus*.

The results of the previously described experiments suggested that proline primarily serves to fuel glutamate synthesis and thus may serve as the major carbon source to fuel gluconeogenesis when glutamate is limiting. In addition, it is predicted that the glucogenic amino acids serine, threonine, glycine, and alanine are primarily catabolized to acetate to fuel ATP synthesis. To address these hypotheses and to determine the fate of carbon following amino acid catabolism, ^13^C-labeled glutamate, arginine, proline, aspartate, histidine, serine, threonine, alanine, and glycine were individually added to CDM and assayed via NMR metabolomics in mid- and post-exponential growth.

### Glutamate, proline, and arginine.

**FIG 4:**
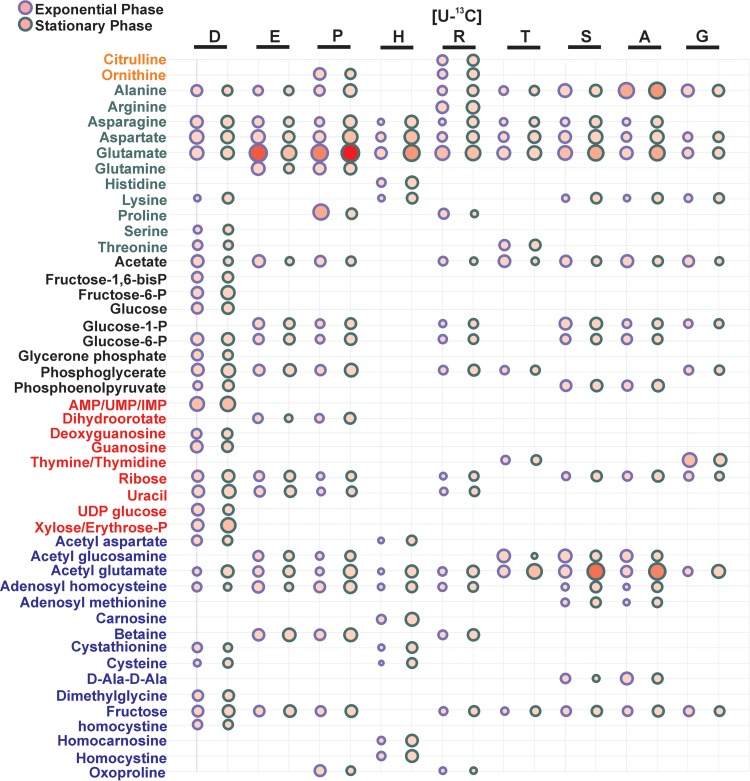
Summary of cellular metabolite concentration changes resulting from supplementation of uniformly labeled amino acids in CDM. The bubble plot summarizes the metabolites derived from (U-^13^C_4_) aspartate (D), (U-^13^C_5_) glutamate (E), (U-^13^C_5_) proline (P), (U-^13^C_6_) histidine (H), (U-^13^C_6_) arginine (R), (U-^13^C_4_) threonine (T), (U-^13^C_3_) serine (S), (U-^13^C_3_) alanine (A), or (U-^13^C_2_) glycine (G) based on an analysis of triplicate 2D ^1^H-^13^C HSQC experiments. Due to the wide dynamic range of NMR intensities, the original NMR peak intensities were transformed by natural logarithm for the purpose of visualization. The relative sizes of the bubbles indicate the transformed values, where a larger diameter indicates a higher NMR peak intensity. The bubbles are colored using a red gradient to represent the original NMR peak intensity, where an increase in color intensity represents a corresponding increase in the original NMR peak intensity. Each level of increase in red intensity corresponds to a 10-fold increase in the NMR intensity. The bubbles with a purple outline identify metabolites observed during exponential phase. Bubbles with a green outline identify metabolites observed during stationary phase. Metabolite names are colored based on the assigned category as follows: orange, urea cycle; green, amino acid; black, glycolysis/gluconeogenesis; red, pentose phosphate pathway/nucleosides; blue, others.

Metabolic intermediates originating from ^13^C-labeled glutamate, proline, and arginine were very similar and included gluconeogenic intermediates (glucose-6P and phosphoglycerate) and intermediates that function in nucleotide biosynthesis (dihydroorotate, ribose, uracil, and adenosyl homocysteine) and glycolipid/lipoteichioic acid synthesis (glucose 1-phosphate) (Fig. 4 and S2A to C) (31). In addition, both aspartate and asparagine were detected from all three ^13^C-labeled amino acids, suggesting that these amino acids were synthesized from oxaloacetate and the TCA cycle. Note that glutamate was synthesized from both proline and arginine, presumably via RocA (Fig. 3 and 4 and S2A to C). However, as previously reported, proline and arginine were not produced from glutamate (22). Importantly, these results suggest that proline and arginine function as carbon sources as they both serve as precursors for glutamate, the key intermediate linking carbon and nitrogen metabolism. However, the amount of glutamate generated from proline was approximately 5-fold greater than the amount generated from arginine (Fig. 4 and S2B and C). And, as previously noted, a mutation in *rocF* had minimal effect on growth in CDM and CDM-E. Note that JE2 contains two copies of arginine deiminase, which can generate citrulline and ornithine from arginine while bypassing RocF (29, 32). However, an *arcA1 arcA2* arginine deiminase mutant or a *rocF arcA1 arcA2* mutant had no significant growth defect in CDM (Fig. S1R and S1S). Thus, these NMR data are consistent with our genotypic growth analysis and show that proline is rapidly catabolized to generate glutamate to facilitate subsequent gluconeogenic reactions.

Previous work demonstrated that arginine is synthesized via proline and the urea cycle in a *ccpA* mutant (22) but not through the well-defined glutamate-to-arginine pathway as described for *B. subtilis* and other bacteria (33). Although genes for the *arg* pathway are present in *S. aureus*, the growth conditions under which they are induced are not known. In a similar manner, work from others (21, 23) demonstrated that arginine served as a precursor for proline biosynthesis in a *ccpA* mutant via RocF, RocD, and ProC. Electrophoretic mobility shift assay (EMSA) analysis demonstrated that CcpA bound to the promoters of both *rocF* and *rocD* but not of *proC* (21). Thus, when *S. aureus* is growing on nonpreferred carbon sources, an intact urea cycle is critical for the interconversion of arginine and proline. Since CDM does not contain a preferred carbon source, it is predicted that CcpA repression would be alleviated and that pathways to interconvert proline and arginine would be transcriptionally active. As predicted, proline synthesis was detected when ^13^C-labeled arginine was added to CDM (Fig. 4B); however, arginine synthesis was not detected from ^13^C-labeled proline. Instead, ornithine was detected from ^13^C-labeled proline, suggesting that, in addition to its use in glutamate synthesis, carbon from proline also enters the urea cycle. To further investigate arginine synthesis in CDM, JE2 and JE2 *ccpA*::*tet* strains were grown in media lacking either proline (CDM-P) or arginine (CDM-R). It was found that the JE2 *ccpA*::*tetL* strain grew in both CDM-P and CDM-R, although growth was delayed in CDM-R (Fig. 1D). In addition, as predicted, the JE2 wild type grew in CDM-P but a *rocF* mutant could not, suggesting that proline synthesis is fueled by arginine in CDM (Fig. 1D). However, unexpectedly, but consistent with the NMR data, JE2 could not grow in CDM-R, suggesting that proline cannot serve as a precursor for arginine synthesis in the absence of a *ccpA* mutation.

### Histidine and aspartate.

Carbon derived from ^13^C histidine was primarily identified in glutamate metabolism and cysteine biosynthesis intermediates (Fig. 4 and S2D). Consistent with our amino acid analysis, where histidine was catabolized more slowly than glutamate, proline, and arginine (Fig. 2), significantly more metabolites (especially glutamate) were identified in the post-exponential phase than in the exponential phase. This observation suggests that histidine is catabolized after proline and arginine are limited or depleted. As the carbon backbone of aspartate enters central metabolism via oxaloacetate, it is not surprising that many metabolic intermediates derived from ^13^C aspartate were identified, including those that function in gluconeogenesis, the pentose phosphate pathway, nucleotide biosynthesis, amino acid biosynthesis, and glutamate metabolism (see Fig. S2E). Indeed, as noted in Fig. S1D, an *aspA* mutant which was unable to synthesize oxaloacetate from aspartate had an increased lag phase compared to the wild type.

10.1128/mBio.01434-16.2 FIG S2  NMR peak intensity of CDM supplemented with ^13^C-labeled amino acids. Metabolites derived from (U-^13^C_5_) glutamate (E) (A), (U-^13^C_5_) proline (P) (B), (U-^13^C_6_) arginine (R) (C), (U-^13^C_6_) histidine (H) (D), (U-^13^C_4_) aspartate (D) (E), (U-^13^C_3_) alanine (A) (F), (U-^13^C_2_) glycine (G) (G), (U-^13^C_4_) threonine (T) (H), or (U-^13^C_3_) serine (S) (I), based on an analysis of the results of triplicate 2D ^1^H-^13^C HSQC experiments. Samples were assayed in either the early exponential phase (OD_600_ = 0.8 to 1.0) or the late exponential phase (OD_600_ = 2.0 to 2.2) of growth. Each experiment was performed in triplicate, and the error bars represent the standard errors of the means. Download FIG S2, PDF file, 0.2 MB. Copyright © 2017 Halsey et al. 2017 Halsey et al. This content is distributed under the terms of the Creative Commons Attribution 4.0 International license.

### Alanine, glycine, threonine, and serine.

**FIG 5:**
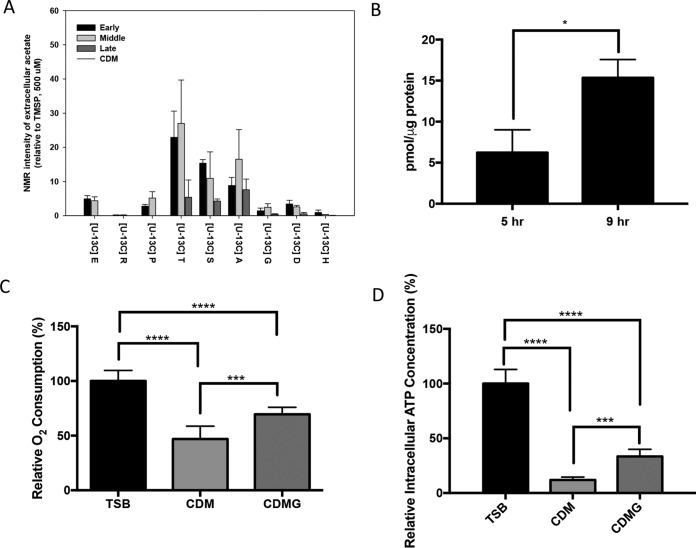
Metabolic indicators of JE2 growth in CDM, TSB, or CDMG. (A) Extracellular acetate detected following aerobic growth in CDM containing (U-^13^C_4_) aspartate (D), (U-^13^C_5_) glutamate (E), (U-^13^C_5_) proline (P), (U-^13^C_6_) histidine (H), (U-^13^C_6_) arginine (R), (U-^13^C_4_) threonine (T), (U-^13^C_3_) serine (S), (U-^13^C_3_) alanine (A), or (U-^13^C_2_) glycine (G). NMR peak intensity was assessed in early exponential (early), mid-exponential (middle), and late exponential (late) growth based on an analysis of the results of triplicate 2D ^1^H-^13^C HSQC experiments. Error bars represent standard errors of the means. (B) Intracellular concentration of citrate as measured in early and post-exponential phases of growth in CDM. (C and D) Relative rate of oxygen consumption (C) and ATP concentration (D) following growth of JE2 in CDM, CDMG, or TSB. Rates of JE2 grown in TSB were set at 100%. Samples for panels C and D were collected in mid-exponential phase. For panels B to D, significance was determined via an unpaired Students *t* test with Welch’s correction (*, *P* < 0.05; ***, *P* < 0.001; ****, *P* < 0.0001).

The metabolic intermediates derived from these ^13^C glucogenic amino acids were very similar, reflecting their direct catabolism to pyruvate (Fig. 4 and S2F to I). Similarly to other amino acids assayed, carbon derived from the glucogenic amino acids was identified in gluconeogenic intermediates, other amino acids, nucleotide biosynthesis, and glutamate. However, significantly more extracellular acetate was derived from alanine, threonine, and serine than from other amino acids (Fig. 5A). Therefore, we hypothesized that a bifurcation exists during amino acid catabolism in which pyruvate generated from the glucogenic amino acids is primarily utilized to generate acetate and ATP via AckA and little carbon enters the TCA cycle via citrate synthase until acetate is consumed from the medium at 6 h of growth. In addition, those amino acids that generate glutamate enter the TCA cycle at 2-oxoglutarate via GudB and serve as a carbon source to subsequently facilitate gluconeogenesis (Fig. 3). Although it is clear from the NMR data that the ^13^C-labeled glucogenic amino acids synthesize glutamate, this hypothesis predicts that mutations within *gltA* (coding for citrate synthase), *acnA* (aconitase), and *icd* (isocitrate dehydrogenase) would not abrogate growth in contrast to mutations in other TCA cycle genes past the 2-oxoglutarate node. Indeed, transposon mutants with mutations within *gltA*, *acnA*, and *icd* were grown in CDM, and, in contrast to the *sucA*, *sdhA*, and *fumC* TCA cycle mutants, growth was observed, albeit with increased lag phase and growth rate and yield defects (Fig. 3 and S1T to V). In agreement with this premise, intracellular citrate concentrations, as an indicator of TCA cycle activity and entry of carbon into the TCA cycle, increased 3-fold during post-exponential growth in CDM compared to exponential growth (Fig. 5B). Collectively, since we detected citrate in early exponential growth in CDM and we observed growth defects with the *gltA*, *icd*, and *acnA* mutants even before acetate was depleted from the medium (6 h of growth; Fig. 1A), these data suggest that the TCA cycle is active throughout growth in CDM but that carbon flux is reduced through the TCA cycle and is instead routed to acetate when amino acids that generate pyruvate are present in the medium. Once these amino acids (primarily serine, threonine, and alanine) are depleted, acetate is consumed and enters the TCA cycle to generate 2-oxoglutarate.

### Respiration is reduced, but is required for viability, during growth of *S. aureus* in CDM.

As an *ackA* mutant was unable to grow in CDM, we hypothesized that the ATP generated by acetate kinase is critical for growth under these conditions. We reasoned that the reducing power generated by the TCA cycle is utilized during gluconeogenesis to regenerate NAD^+^ and maintain redox balance. Thus, the net level of NADH may be reduced during growth in CDM below the levels needed to drive oxidative phosphorylation from the staphylococcal NADH:quinone oxidoreductase. Therefore, experiments were designed to determine if respiration is reduced during growth in CDM compared to TSB or CDM containing 14 mM glucose (CDMG). The relative rate of oxygen consumption during growth in CDM was approximately 50% of the rate in TSB, and the rate was significantly lower than that detected in CDMG (Fig. 5C). Consistent with these observations, the relative intracellular ATP concentration during growth in CDM was 12% of the concentration in TSB (Fig. 5D). Collectively, these data suggest that growth in CDM yields significantly decreased respiratory activity and coincident ATP synthesis.

**FIG 6:**
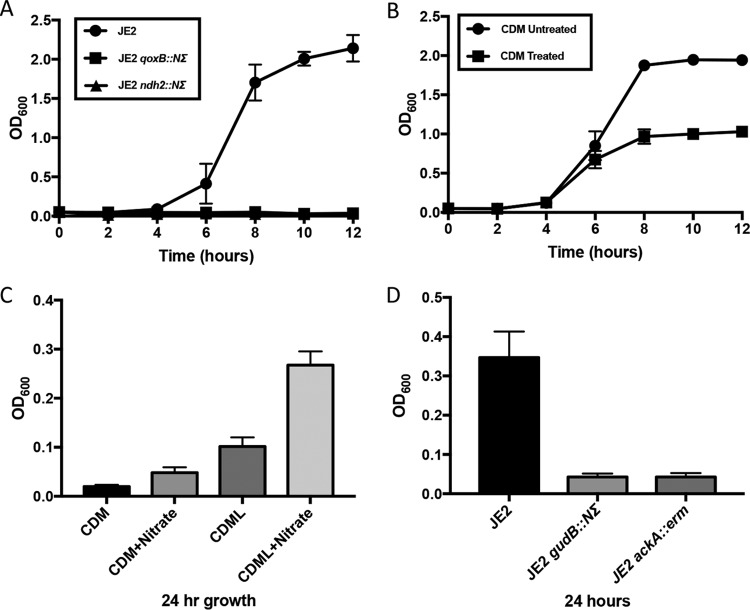
Importance of aerobic and anaerobic respiration during growth in CDM. (A) Aerobic growth of JE2, JE2 *qoxB*::*NΣ*, and JE2 *nrd2*::*NΣ* strains in CDM. (B) Growth of JE2 in CDM following the addition of the ATP synthase inhibitor DCCD in early exponential phase. (C) Anaerobic growth (24 h) of JE2 in CDM containing either 3 mM sodium nitrate or 0.5% lactate (CDML). (D) Anaerobic growth (24 h) of JE2, JE2 *gudB*::*NΣ*, and JE2 *ackA* strains grown in CDM containing sodium nitrate (3 mM) and lactate (0.5%). Growth curve analyses were performed in three independent experiments; error bars represent the standard errors of the means (SEM).

*S. aureus* contains a branched respiratory chain and has two terminal menaquinol oxidases, QoxABCD (34, 35) and CydAB (36). QoxABCD is promiscuous and can function as both a cytochrome *aa_3_*-type and *bo*_*3*_-type oxidase, whereas CydAB is a *bd*-type oxidase (37). Respiratory complexes that feed the menaquinone pool include succinate dehydrogenase (Sdh) (38), Lqo (4, 6, 39), and malate quinone oxidoreductase (Mqo) (4, 6). Furthermore, *S. aureus* does not contain a complex I NADH oxidoreductase but instead encodes a type 2 nonelectrogenic NADH:quinone oxidoreductase (Ndh2) that may be linked to MpsA, a NuoL-like protein that functions to translocate cations but has no NADH binding capability (40). The lack of growth demonstrated by a succinate dehydrogenase mutant (Fig. 3 and S1L) and the decreased ATP concentration and oxygen consumption noted during growth in CDM led us to address the importance of the electron transport chain during growth in CDM. Transposon mutants deficient in NADH:quinone oxidoreductase (coded for by *nrd*; SAUSA300_0844) and quinol oxidase (*qox*) were grown in CDM; both the *nrd* mutant and the *qox* mutant were unable to grow aerobically in CDM (Fig. 6A). Furthermore, growth of *S. aureus* was halted in CDM when the ATP synthase inhibitor DCCD (*N*,*N*′-dicyclohexylcarbodiimide) was added to the medium in early exponential phase (Fig. 6B). In contrast, DCCD had little effect on growth in TSB or CDMG (see Fig. S3A and B). Collectively, the results of these experiments suggest that ATP generated via both substrate-level phosphorylation and oxidative phosphorylation is required for growth in CDM and that the importance of the PTA-AckA pathway under these conditions is most likely linked to the reduced rate of respiration. Note that a *cydA* mutant had no growth phenotype, suggesting that the cytochrome *bd* oxidase does not have a recognizable function during growth in CDM (see Fig. S1W).

10.1128/mBio.01434-16.3 FIG S3  Growth of JE2 following addition of DCCD inhibitor. (A and B) Growth of JE2 in TSB (A) or CDMG (B) following the addition of the ATP synthase inhibitor DCCD in the early exponential phase. Data were collected from results of three independent experiments; error bars represent the standard errors of the means (SEM). Download FIG S3, TIF file, 1.4 MB. Copyright © 2017 Halsey et al. 2017 Halsey et al. This content is distributed under the terms of the Creative Commons Attribution 4.0 International license.

### Growth of *gudB* and *ackA* mutants in CDM is impaired under conditions of anaerobic growth.

The infection model proposed by Spahich and colleagues suggests that *S. aureus* growth under conditions of mild NO stress within an anoxic, glucose-depleted abscess requires both lactate and peptides. Thus, we assessed whether *gudB* and *ackA* were also required for growth in CDM under anaerobic growth conditions. JE2 was grown in CDM with or without lactate and/or nitrate, which was used as a terminal electron acceptor. These experiments demonstrated that enhanced growth was observed only in CDM containing lactate and nitrate as previously described (4, 6) (Fig. 6C). Thus, JE2 *gudB*::*NΣ* and JE2 *ackA* strains were grown in CDM containing lactate and nitrate. As predicted, no growth was observed for either of these mutants (Fig. 6D).

### *S. aureus* does not utilize proline or arginine during growth in CDMG.

**FIG 7:**
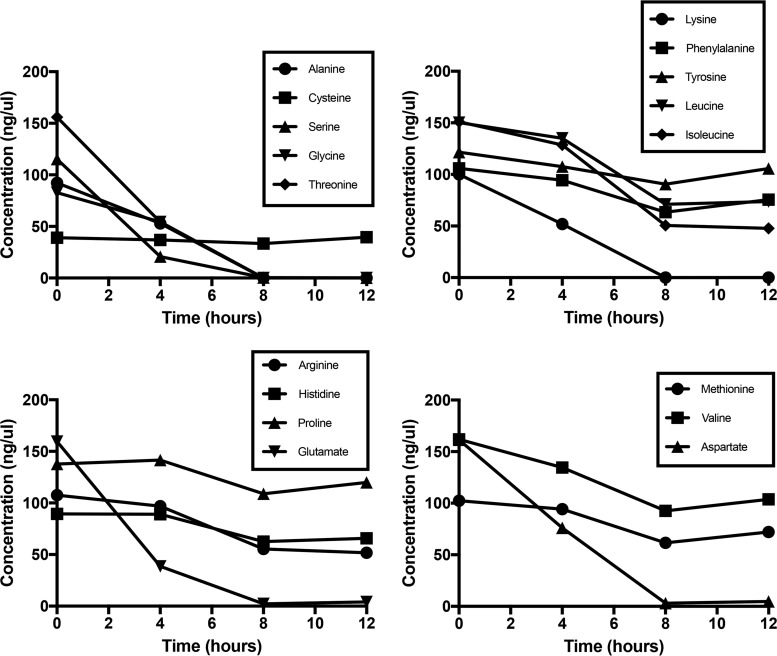
Amino acid consumption of JE2 following growth in CDMG. JE2 was grown aerobically in CDMG, and amino acid concentrations (mM) were measured from the supernatant at 0, 4, 8, and 12 h of growth. The results are representative of two independent experiments.

During our initial studies of growth in CDM, we noted several differences when 14 mM glucose was added to the medium. First, aerobic growth of *S. aureus* JE2 in CDMG reached an OD_600_ maximum of approximately 5.5 following 6 h of growth, at which time glucose was exhausted from the medium (see Fig. S4A). In contrast to the results seen with growth in TSB (28) and CDM (Fig. 1A), acetate was not completely consumed from CDMG, suggesting that growth arrest and subsequent acetate oxidation via the TCA cycle may be halted due to depletion of critical amino acids or other C4/C5 compounds as previously discussed (38), although the exact mechanisms are not clear (see Fig. S4A). Amino acid analysis of spent medium demonstrated that aspartate, glutamate, lysine, and the glucogenic amino acids alanine, serine, glycine, and threonine were rapidly depleted from the medium (Fig. 7). However, the remaining amino acids, including arginine, histidine, and proline, that are known to generate glutamate during growth in CDM were incompletely utilized following 12 h of growth. In fact, a mutation in *gudB* had little effect on growth in CDMG, in contrast to that observed with growth in CDM (see Fig. S4B). As discussed previously, investigations have shown that arginine and proline catabolism is repressed via CcpA and carbon catabolite repression (21, 22). However, following glucose consumption at 6 h of growth, rapid arginine and/or proline catabolism was not observed. Furthermore, no growth was observed in CDMG when proline (CDMG-P) or arginine (CDMG-R) was subtracted from the medium (see Fig. S4C). In contrast, a lack of glutamate in CDMG (CDMG-E) did not result in a significant growth defect (see Fig. S4C). NMR analysis performed using ^13^C_6_-labeled glucose confirmed that glucose was metabolized to synthesize glutamate, thus facilitating growth in CDMG-E (see Fig. S5). However, almost undetectable levels of ^13^C-labeled arginine or proline were detected during growth with CDMG containing ^13^C_6_-labeled glucose, confirming previous data demonstrating that arginine and proline need to be acquired from exogenous sources when *S. aureus* grows on preferred carbon sources such as glucose (21, 22). Collectively, these data suggest that growth of *S. aureus* in CDMG results in rapid utilization of glucose, serine, threonine, alanine, glycine, glutamate, lysine, and aspartate. However, proline and arginine, key amino acids that fuel glutamate synthesis under conditions of growth in CDM, are not utilized as a carbon source when glucose is exhausted from CDMG. Both proline and arginine are required for growth in CDMG for utilization during protein synthesis, however, as glutamate does not serve as a substrate for their synthesis. Lastly, it is unclear how lysine is rapidly utilized during growth in CDMG, as pathways that function to catabolize lysine were not identified in *S. aureus* FPR3757 (see Table S1).

10.1128/mBio.01434-16.4 FIG S4  Growth of *S. aureus* JE2 in CDMG. (A) Aerobic growth of JE2 in CDM displaying glucose consumption and acetate, ammonia, and lactate production. Data are representative of experiments performed three independent times. (B and C) Growth of JE2 and JE2 *gudB*::*NΣ* strains in CDMG (B) and of JE2 in CDMG, CDMG lacking arginine (CDMG-R), CDMG lacking glutamate (CDMG-E), and CDMG lacking proline (CDMG-P) (C). Data in panel B are representative of results of two independent experiments, whereas data in panel C are representative of experiments performed in triplicate; error bars represent the standard errors of the means. Download FIG S4, TIF file, 1.7 MB. Copyright © 2017 Halsey et al. 2017 Halsey et al. This content is distributed under the terms of the Creative Commons Attribution 4.0 International license.

10.1128/mBio.01434-16.5 FIG S5  NMR peak intensity of CDMG supplemented with ^13^C-labeled glucose. Metabolites were derived from (U-^13^C_6_) glucose following aerobic growth in CDMG based on an analysis of triplicate 2D ^1^H-^13^C HSQC experiments. Metabolites were assayed in early exponential, mid-exponential, or late exponential growth. Each experiment was performed in triplicate, and the error bars represent the standard errors of the means. Download FIG S5, TIF file, 2.1 MB. Copyright © 2017 Halsey et al. 2017 Halsey et al. This content is distributed under the terms of the Creative Commons Attribution 4.0 International license.

### *putA*, *rocD*, *hutU*, *rocA*, *gudB*, and *rocF* are regulated via carbon catabolite repression and CcpA.

**FIG 8:**
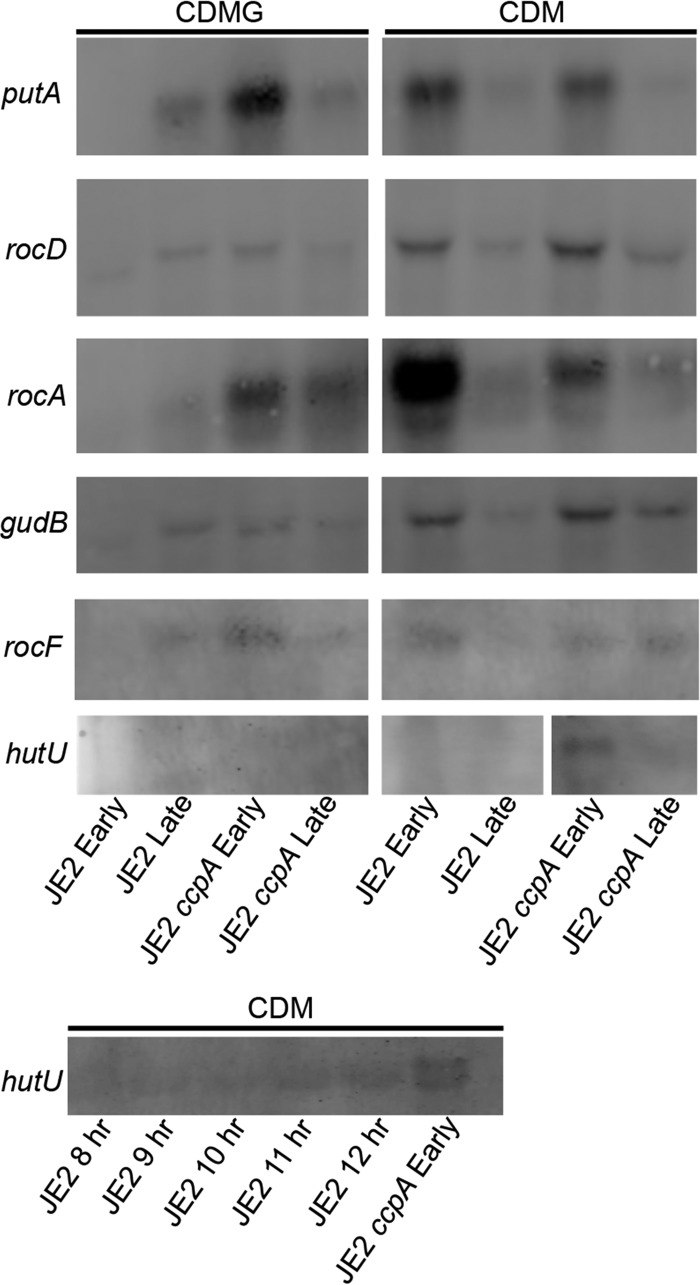
Transcriptional analysis of *putA*, *rocD*, *rocA*, *gudB*, *rocF*, and *hutU* following growth in CDM and CDMG. Northern analysis was performed with RNA isolated from exponential (early) and post-exponential (late) phases of growth (both CDM and CDMG) from JE2 and JE2 *ccpA*::*tetL* strains. Equal loading of RNA was ensured via staining of the RNA gel with ethidium bromide and visualization of the 16S and 23S rRNA (data not shown).

The results of the studies described above suggest that proline and arginine catabolism is dependent upon carbon catabolite repression. Indeed, previous studies demonstrated that the interconversion of proline and arginine is repressed via CcpA at *rocF*, *rocD*, and* putA* in the presence of glucose (21, 22), indicating that these amino acids (which can be converted to glutamate) can be used as alternative carbon sources. Therefore, it was hypothesized that the conversion of histidine to glutamate, in addition to the conversion of arginine and proline to glutamate via pyrolline-5 carboxylate and RocA and the subsequent conversion of glutamate to 2-oxoglutarate via GudB, would also be CcpA repressed, as these reactions would be necessary only if there were no other preferred carbon sources available (Fig. 3). Therefore, Northern blots were utilized to assess transcription of *putA*, *rocD*, *hutU*, *rocA*, *gudB*, and *rocF* in both JE2 and a JE2 *ccpA*::*tetL* mutant following growth in CDMG and CDM. These experiments revealed that transcription of *putA*, *rocD*, *rocA*, *gudB*, and *rocF* was induced after glucose was exhausted from CDMG (Fig. 8). In addition, as expected, transcription of *putA*, *rocD*, *rocA*, *gudB*, and *rocF* in exponential growth was *ccpA* dependent, as transcription was detected in the JE2 *ccpA*::*tetL* mutant following exponential growth in CDMG. Furthermore, as predicted, transcription of *putA*, *rocD*, *rocA*, *gudB*, and *rocF* was detected during exponential growth in CDM. Transcription of *hutU*, in contrast, was detected only in the JE2 *ccpA*::*tetL* mutant in the exponential phase of growth and not in the exponential phase of growth of wild-type JE2. On the basis of the NMR and amino acid utilization data (Fig. 2 and 4), we predicted that *hutU* transcript would be detected in the post-exponential-growth phase following proline and arginine depletion in CDM, but we were unable to detect this transcript during our initial studies. In addition, *hutU* transcript was not detected in CDMG, even in the *ccpA* mutant. These data may suggest that growth arrest occurs in CDMG before potentially multiple regulatory networks induce *hutU* expression. Indeed, histidine catabolism in *B. subtilis* is regulated via both CcpA and CodY (41, 42). Therefore, to further assess *hutU* transcription, RNA was collected during growth in CDM from 8 to 12 h. Note that *hutU* transcript was detected at both 11 and 12 h of growth (Fig. 8), which is consistent with the NMR (Fig. 4 and S2D) and amino acid utilization (Fig. 2) data demonstrating that histidine is catabolized only when proline and arginine are limited. Collectively, these data demonstrate that pathways that function to fuel glutamate catabolism, and *gudB*, which fuels the TCA cycle from glutamate, are all regulated by CcpA and are induced only when glucose is not present in the medium.

## DISCUSSION

Our knowledge of how *S. aureus* proliferates within multiple niches of the host is limited. Nevertheless, recent studies have found that *S. aureus* can adapt to changing levels of carbon sources and oxygen availability found within specific organ systems. For example, Hammer and colleagues determined that *S. aureus* bacteria defective in aerobic respiration were unable to mediate infection in the heart or liver but that colonization and abscess formation in the kidney were unaffected (35). Complementing these findings, *S. aureus* mutants defective in anaerobic fermentation were less virulent than the wild type in a mouse model of kidney infection (5). Additionally, mutants defective in CcpA were less effective at infecting the liver where glucose was abundant, but the infection burden in the kidney was not affected (21). Thus, taken together, these data suggest that *S. aureus* uses distinct central metabolic pathways to adapt to the different carbon or oxygen sources that are available within the organ system or site of infection.

An elegant model has emerged demonstrating how *S. aureus* survives within a staphylococcal abscess (6). The antimicrobial action of NO occurs at multiple nodes of central metabolism, including the TCA cycle and respiration. However, the NO concentration at the center of staphylococcal abscesses is thought to be below 1 μM, allowing respiration and other metabolic processes to be functional (15, 43). Furthermore, glucose is depleted due to phagocytic cell activity, whereas lactate is abundant due to excretion during staphylococcal growth (6, 16). Thus, the major carbon sources for *S. aureus* at the center of abscesses are lactate and peptides. Lactate is utilized via lactate quinone oxidoreductase (Lqo), which generates pyruvate and subsequently acetate via Pta/AckA, whereas peptides provide a carbon source for gluconeogenesis. The goal of the study was to assess amino acid catabolism and to determine the importance of particular amino acid catabolic pathways during growth without glucose or other preferred carbon sources.

Bioinformatic analyses of USA300 LAC (32) found that *S. aureus* has the potential ability to catabolize 11 amino acids to pyruvate, oxaloacetate, or 2-oxoglutarate (see Table S1 in the supplemental material). It should be noted that these pathways are predicted and that novel pathways could be present in *S. aureus*. However, growth in CDM and subsequent amino acid analysis verified the predictions for 10 of the 11 amino acids. Our results are similar to those of previously published studies assessing amino acid catabolism or utilization in *S. aureus*, although the media used varied slightly in these studies (44–46). The lone amino acid not catabolized, but predicted based on bioinformatics analyses, was cysteine. This result may have occurred because CDM contains MgSO_4_ as a sulfur source. It is well established that *S. aureus* cannot utilize sulfate, sulfite, or sulfonates as the source of sulfur in cysteine biosynthesis, whereas it can use thiosulfate, sulfide, or glutathione (47). Thus, we predict that a feedback mechanism exists for repressing cysteine catabolism if an appropriate sulfur source for cysteine synthesis is not present. In addition, we observed that lysine was catabolized when *S. aureus* was grown in CDMG but not CDM. Some species are known to ferment lysine to acetate (48), but metabolic pathways to perform this function are not immediately recognized in the *S. aureus* genome.

Glutamate is the major amino donor for most anabolic enzymatic reactions and yet also functions as a key intermediate in carbon metabolism (49, 50). Our results suggest that glutamate, and those amino acids that can be converted into glutamate, serves as a central carbon source to facilitate growth in media lacking a preferred carbon source such as glucose. First, glutamate dehydrogenase (GudB), an enzyme that catalyzes the conversion of glutamate to 2-oxoglutarate, is required for growth in CDM. In addition, all enzymes (excluding Mqo1, which is rescued by anaplerotic reactions generating oxaloacetate) that function in the TCA cycle downstream of 2-oxoglutarate, including phosphoenolpyruvate carboxykinase (PckA), which generates PEP from oxaloacetate, are required. Thus, glutamate fuels the TCA cycle allowing subsequent gluconeogenic reactions and presumably serves as the major amino group donor to cellular reactions.

Second, although NMR analysis found that ^13^C carbon from all of the amino acids tested eventually was incorporated into glutamate, only mutations in those genes that code for enzymes that function to generate glutamate from proline resulted in a significant growth defect. Other than arginine, histidine, and proline, all amino acids must generate glutamate via the TCA cycle and glutamate synthase (GOGAT). However, a mutation in *gltB* (encoding a large subunit of glutamine oxoglutarate aminotransferase [GOGAT]; see Fig. S1X in the supplemental material) conferred no growth defect in CDM, suggesting that the importance of glutamate synthesis from amino acids other than proline and histidine is minimal. As indicated by NMR, arginine can also be directly converted to glutamate by the arginase pathway (RocF-RocD-RocA) (Fig. 3). Alternatively, independently of RocF, arginine deiminase (29) can synthesize ornithine from arginine, which can then be catabolized to glutamate via RocD and RocA. However, individual *rocF* (see Fig. S1F) and *arcA1 arcA2* (see Fig. S1R) mutants did not have a growth defect in CDM, whereas a *rocF arcA1 arcA2* (see Fig. S1S) mutant had only a post-exponential growth defect, suggesting that arginine catabolism does not significantly contribute to exponential growth. However, it was surprising that a *rocD* mutant had a growth phenotype in CDM similar to that of the *rocA* mutant (see Fig. S1Y and Z); RocD converts ornithine, which can be synthesized from arginine via RocF, to P5C (1-pyrroline-5-carboxylic acid) (Fig. 3). Since the *rocF* (see Fig. S1F) mutant did not have a significant growth defect, one would predict that a *rocD* mutant would phenocopy a *rocF* mutant. *S. aureus* also encodes a nitric oxide synthase (*nos*), which catalyzes the formation of citrulline from arginine, generating NO (51). Thus, it is possible that arginine is primarily catabolized via NOS and not arginase or arginine deiminase during growth in CDM, but whether this is the case is currently unknown. Nevertheless, complementing these genetic findings, comparison of NMR peak intensities suggested that the level of glutamate synthesized via proline was approximately 5 times and 6 times that synthesized from arginine in the exponential and post-exponential phases, respectively, and was approximately 5 times and 2.5 times that synthesized from histidine in the exponential and post-exponential phases, respectively (Fig. 4). Thus, as our NMR and amino acid utilization data demonstrate, histidine is utilized after proline levels become limiting. Collectively, our data suggest that proline is the primary substrate for glutamate synthesis during exponential phase and that histidine is utilized once proline is limiting. Furthermore, arginine is a minor source of glutamate synthesis in both the exponential and post-exponential phases of growth.

Lastly, we found that the entire metabolic pathway that functions to catabolize both proline and arginine is under carbon catabolite and CcpA control. Thus, proline, histidine, and arginine are catabolized only when glucose is limiting. However, why is proline the primary source of glutamate when *S. aureus* is growing without glucose? We have previously proposed a model whereby arginine is rapidly depleted within an abscess due to its preferential uptake by host innate immune cells for inducible nitric oxide synthase (iNOS) (52), arginase (53, 54), and cell-mediated (55) immunity, effectively reducing the amount of free extracellular arginine available in the microenvironment. Previous studies have shown significant induction of both iNOS and arginase expression during *S. aureus* abscess formation (22), further suggesting that arginine depletion occurs at the infection site. Although nothing is known regarding the concentration of free proline or histidine in a staphylococcal infection, proline is the second-most-abundant amino acid found in collagen, which is the most abundant protein in animals (56). Indeed, collagen is a major constituent of the fibrotic wall surrounding a staphylococcal abscess (22). We hypothesize that *S. aureus* uses a combination of host-derived proteases, known to be induced to facilitate tissue remodeling during abscess formation (57, 58), and its own proteases that are known to degrade collagen (59–61) to liberate free proline or proline-containing peptides that are subsequently utilized by *S. aureus* to synthesize arginine and glutamate. Thus, since proline is likely to be present at high concentrations in abscesses and can be transformed to both arginine and glutamate, it is proposed that *S. aureus* has evolved to rapidly synthesize both glutamate and arginine from proline when preferred carbon sources such as glucose are lacking.

We also propose that catabolism of the glucogenic amino acids (e.g., serine, threonine, and alanine) during growth in CDM functions primarily to generate acetate and ATP via substrate-level phosphorylation. Several observations suggested that conversion of acetyl-CoA to 2-oxoglutarate is most active in the post-exponential phase during growth in CDM. First, acetate consumption, as an indicator of TCA cycle activity, is observed during the post-exponential phase (Fig. 1A). Second, citrate concentrations, another indicator of TCA cycle activity, increase significantly in the post-exponential phase during growth in CDM (Fig. 5A). Third, in contrast to the results seen with TCA cycle enzymes that function to generate oxaloacetate from 2-oxoglutarate, mutations in *gltA*, *acnA*, or *icd* do not abrogate growth. Thus, we propose that the glucogenic amino acids are catabolized to acetate via Pta/AckA and secreted. The acetate is subsequently catabolized via the TCA cycle in the post-exponential phase since acetate represents a secondary carbon source. This supposition is supported by the findings indicating that ^13^C-labeled glutamate was primarily detected in the post-exponential phase when CDM was supplemented with ^13^C-labeled alanine, serine, or threonine. Glutamate can be synthesized from these amino acids only via TCA cycle activity and generation of glutamate via GOGAT and 2-oxoglutarate.

Why is a mutation in *ackA* deleterious to growth in CDM? Although a mutation in *ackA* is detrimental to growth in TSB, it does not completely abrogate growth but instead enhances glycolytic flux and diverts carbon to the TCA cycle (27). We first hypothesized that the ATP generated via AckA was fundamentally important due to a decrease in ATP generation via the respiratory chain during growth in CDM. However, although we found that oxygen consumption, ATP synthesis, and the NAD^+^/NADH ratio are significantly decreased during growth in CDM compared to TSB, we found that mutations in NADH dehydrogenase and quinol oxidase were also deleterious to growth. In addition, time-kill curve experiments performed using the ATP synthase inhibitor DCCD halted growth in CDM whereas growth was unaffected in TSB and CDMG. Thus, we propose that the ATP generated from both AckA and ATP synthase via respiratory activity is important for growth in CDM. Indeed, amino acid transport requires ATP and is a more energy-intensive process than carbohydrate transport, which is facilitated through the phosphotransferase (PTS) system. Supporting this notion, anaerobic growth of JE2 in CDM required both lactate and nitrate and was *gudB* and *ackA* dependent. These data suggest that glutamate catabolism, substrate-level phosphorylation, and ATP derived from anaerobic respiration were all required for growth in an anaerobic environment which mimicked the anoxic environment found in an abscess (Fig. 6C). Alternatively, the lack of growth in CDM with an *ackA* mutant may be linked to recycling of coenzyme A, which is required for the conversion of 2-oxoglutarate and succinyl-CoA via SucA (62). However, it is unclear how a mutation in *ackA* would affect activity of Pta, which functions to generate acetyl phosphate while regenerating CoA. Additionally, recent studies have documented that an *ackA* mutant generates a redox imbalance in *S. aureus* (26). Thus, the growth phenotype observed with the *ackA* mutant may be multifactorial.

Finally, multiple pathways fueling glutamate catabolism (synthesis from proline, arginine, and histidine) are all under the control of repression of CcpA and thus are active only when preferred carbon sources such as glucose are depleted. This provides a rapid mechanism to facilitate growth using amino acids as carbon sources when glucose is not available. However, it is unclear why growth of JE2 in CDM-R is dependent upon a mutation in *ccpA*. One would expect that CcpA repression would be alleviated during growth in CDM without a preferred carbon source available. However, we did not observe growth of JE2 in CDM-R, and growth of the JE2 *ccpA*::*tetL* mutant in CDM-R was dependent upon *putA*, suggesting that proline was the source of arginine, as previously described (22). This observation may suggest that regulation of the urea cycle may involve multiple layers of regulation, including CcpA regulation. In addition, GudB in *B. subtilis* is upregulated by the presence of proline, arginine, or ornithine (63); thus, it is possible that *gudB* transcription requires arginine, at least in the presence of CcpA, to be fully functional. However, it should be noted that slight growth of JE2 in CDM-R was observed after 24 to 30 h of growth, but the significance of this observation is not clear (data not shown).

In conclusion, we have found that glutamate, and those amino acids that serve to fuel its synthesis, are central carbon sources during growth in medium lacking glucose. In addition, the glucogenic amino acids are catabolized to generate acetate and produce ATP via AckA; the ATP generated via this reaction is critical for growth in medium lacking glucose such as CDM. Further work is required to determine the function and expression of these catabolic pathways to determine their importance *in vivo*.

## MATERIALS AND METHODS

### Bacterial strains and culture conditions.

The *S. aureus* strains used in this study were all derived from JE2 (30). Defined *bursa aurealis* transposon mutants were obtained from the Nebraska Transposon Mutant Library and backcrossed to JE2 using Φ11 (30). The JE2 *ccpA* and JE2 *ackA* strains have been previously described (22, 27, 64). *S. aureus* strains were grown in tryptic soy broth (TSB; Becton, Dickinson, Franklin Lakes, NJ); complete defined medium (CDM), as previously described (25), except no glucose was added; or CDM with 0.25% glucose (CDMG). Cultures were grown aerobically in a 10:1 flask-to-volume ratio, with shaking at 250 rpm at 37°C. *S. aureus* cultures were inoculated to an optical density at 600 nm (OD_600_) of 0.05 from washed (phosphate-buffered saline [PBS]) overnight cultures grown in TSB, CDM, or CDMG. Bacterial growth was assessed by measuring the optical density at 600 nm. Anaerobic growth was measured in an anaerobic chamber (Coy Laboratory Products, Inc., Grass Lakes, MI) using a 10:1 flask-to-volume ratio in CDM supplemented with 1.0 g/liter l-cysteine hydrochloride (Sigma) with or without the addition of l-lactate (0.5%) and/or sodium nitrate (20 mM). Growth was assessed in the anaerobic chamber with shaking at 37°C and 250 rpm to ensure that the cells did not settle in the flask. For time-kill curve experiments using ATP synthase inhibitors, 100 μM DCCD (*N*,*N*′-dicyclohexylcarbodiimide)–95% ethanol was added to cultures during early exponential growth (OD_600_ = 0.5 for CDM and 1.0 for TSB and CDMG). The *putA* (pNF275) and *gudB* (pNF311) complement plasmids were constructed using pBK123 (65). *putA* was cloned into the PstI and BamHI restriction sites of pBK123 using primers 2344 and 2345 (see Table S2 in the supplemental material), whereas *gudB* was cloned into the Pst1/BamHI restriction sites using primers 2846 and 2848 (see Table S2).

10.1128/mBio.01434-16.7 TABLE S2  Primers used in study. Restriction sites are denoted in italics. Download TABLE S2, DOCX file, 0.1 MB. Copyright © 2017 Halsey et al. 2017 Halsey et al. This content is distributed under the terms of the Creative Commons Attribution 4.0 International license.

### Measurement of bacterial metabolites.

Bacterial cultures were collected (1-ml aliquots) and centrifuged at 14,000 rpm for 3 min. The supernatants were removed and stored at −80°C until use. Acetic acid, glucose, ammonia, and d- and l-lactic acid concentrations were determined according to the instructions of the manufacturer using kits purchased from R-Biopharm.

### Measurement of TCA cycle and respiration activity.

For all assays, JE2 was grown overnight aerobically in 25 ml of appropriate media. Cultures were inoculated to an OD_600_ of 0.05 in TSB, CDM, or CDMG and were grown to early exponential phase (OD_600_ = 0.5 for CDM and 1.0 for TSB and CDMG).

### ATP concentration.

Intracellular ATP concentrations were determined using a BacTiter-Glo kit (Promega) according to the manufacturer’s protocol and normalized to total optical units.

### NAD^+^/NADH concentration.

Aliquots of bacterial cultures (24 ml) were harvested by centrifugation at 4°C for 10 min at 4,000 rpm. Intracellular NAD^+^ and NADH concentrations were determined using a fluorescent NAD^+^/NADH kit (Cell Technology) as previously described (27) and normalized to total cellular protein concentrations.

### Oxygen consumption.

Samples were collected and diluted in air-saturated TSB, CDM, or CDMG to an OD_600_ of 0.3. Relative oxygen consumption rates were determined for up to 120 min at 37°C by using a MitoXpress oxygen-sensitive probe (Luxcel Biosciences) according to the manufacturer’s instructions and normalized to total optical units.

### Citrate concentrations.

Aliquots of bacterial cultures (24 ml) were harvested by centrifugation at 4°C for 10 min at 4,000 rpm. The bacterial pellets were washed twice with 1 ml of phosphate-buffered saline (pH 7.4) (PBS) and subsequently resuspended in 0.5 ml PBS, followed by the addition of 0.1 ml ice-cold 3 M perchloric acid. The cells were lysed using lysing matrix B tubes in a FastPrep FP120 instrument (Qbiogene, Inc.). The lysates were centrifuged for 3 min at 14,000 rpm. Subsequently, 300 μl of supernatant was neutralized with 75 μl of saturated solution of potassium bicarbonate and centrifuged at 4°C for 3 min at 14,000 rpm. Intracellular citrate concentrations in the lysates were determined by using a citrate fluorometric assay kit (BioVision) according to the manufacturer’s protocol and normalized to total cellular protein concentrations.

### Amino acid analysis.

JE2 was grown overnight in 50 ml (500-ml flask) of CDMG. Cultures were inoculated to an OD_600_ of 0.05 in CDM or CDMG and grown for 12 h. One milliliter of media was collected every 4 h and pelleted for 3 min at 14,000 rpm. Supernatant was collected and filtered through Amicon Ultra centrifugal filters (Millipore) (3,000 molecular weight cutoff [MWCO]) according to the manufacturer’s protocol. Amino acid analysis was performed by the Protein Structure Core Facility, University of Nebraska Medical Center (UNMC), using a Hitachi L-8800 amino acid analyzer.

### NMR sample preparation.

Three independent 50-ml cultures of *S. aureus* JE2 were grown to exponential phase (OD_600_ = 0.8 to 1.0) and post-exponential/stationary phase (OD_600_ = 2.0 to 2.2) in CDM containing ^13^C_5_-labeled glutamate, ^13^C_5_-labeled proline, ^13^C_6_-labeled arginine, ^13^C_6_-labeled glucose, or ^13^C_3_-labeled pyruvate (Sigma-Aldrich). Cultures were normalized to an OD_600_ of 40 and pelleted by centrifugation (6,000 rpm at 4°C for 5 min). Pellets were subsequently washed two times with 10 ml of cold sterile water and resuspended in 1 ml cold sterile water and transferred to a lysing matrix B tube (MP Biomedicals). The pellet was lysed using an FP120 FastPrep cell disrupter (MP Biomedicals) and centrifuged at 13,200 rpm for 15 min at 4°C. The pellet was reextracted by the use of 1 ml cold sterile water. Supernatants from the two extractions were combined and stored at −80°C for NMR analysis.

### NMR data collection and analysis.

The collected supernatants were lyophilized and reconstructed in NMR buffer (KH_2_PO_4_/K_2_HPO_4_ buffer–D_2_O [pH 7.4] [uncorrected], with 500 μM trimethylsilylpropanoic acid [TMSP] as an internal standard). Two-dimensional (2D) ^1^H-^13^C heteronuclear single-quantum coherence (HSQC) spectra were collected on a Bruker DRX Avance 500-MHz spectrometer equipped with a 5-mm-long triple-resonance cryoprobe (^1^H, ^13^C, and ^15^N) with a *z* axis gradient, a BACS-120 sample changer, Bruker IconNMR, and an automatic tuning and matching (ATM) unit. NMRPipe (28) and NMRViewJ (29) were used to process and analyze the collected spectra. The TMSP internal standard was used for chemical shift referencing and normalization of NMR peak intensities. NMR peaks from the 2D ^1^H-^13^C HSQC spectra were annotated by comparing the observed ^1^H and ^13^C chemical shifts to the metabolite reference data from the Platform for RIKEN metabolomics (PRIMe; http://prime.psc.riken.jp/) (30), Human Metabolome Database (HMDB; http://www.hmdb.ca/) (31), Madison metabolomics Consortium Database (http://mmcd.nmrfam.wisc.edu/) (32), Metabominer (http://wishart.biology.ualberta.ca/metabominer/) (33), and BiomagResBank (BMRB; http://www.bmrb.wisc.edu//) (34) with error tolerances of 0.08 ppm and 0.25 ppm for ^1^H and ^13^C chemical shifts, respectively. The relative intensity (i.e., concentration) of each metabolite was calculated by averaging the intensities of all NMR peaks unambiguously assigned to the metabolite.

### RNA isolation and Northern blot analysis.

Cultures of *S. aureus* JE2 and JE2 *ccpA* strains were grown overnight in CDM and CDMG and inoculated to an OD_600_ of 0.05 into TSB (TSB overnight), CDM, and CDMG, respectively. Cultures were grown at 37°C in a 1:10 vol-to-flask ratio at 250 rpm. Fifteen milliliters of bacterial culture was collected at the mid-exponential phase (3 h to 6 h) and the post-exponential phase (7 h to 10 h) of growth and pelleted at 5,000 rpm for 10 min at 4°C. Pellets were resuspended in RLT buffer (Qiagen, Inc.) with 1% β-mercaptoethanol and transferred to lysing matrix B tubes (MP Biomedicals). They were then processed in an FP120 FastPrep cell disrupter (MP Biomedicals) for 24 s at a setting of 6.0. Cells were then pelleted at 13,000 rpm for 15 min at 4°C. The top phase was combined with 500 μl of ethanol, and the samples were then processed using an RNeasy minikit, according to the instructions of the manufacturer (Qiagen, Inc.) Primers listed in Table S2 were used to make DNA probes, which were subsequently labeled with digoxigenin (DIG)-labeled dUTP (Roche). One microgram of RNA was used for Northern analysis, which was performed using DIG buffers and washes (Roche). Anti-digoxigenin-AP Fab fragments (Roche) was used with ECF substrate (GE Healthcare) for detection. Blots were visualized using a Typhoon FLS 7000 imaging system (GE Healthcare).
