# Supplementary material for: Amino Acid Catabolism in Staphylococcus aureus and the Function of Carbon Catabolite Repression
Source: mBio. 2017 Feb 14;8(1):e01434-16. doi: 10.1128/mBio.01434-16 (PMC5312079; doi:10.1128/mBio.01434-16)
Supplement: TABLE S1 [file mbo001173181st1.docx]

**Table S1. Predicted amino acid catabolism in *Staphylococcus aureus* FPR3757^a^**

**Amino acids yielding pyruvate**

**Amino acid Reaction Enzyme FPR3757**

Alanine (1) Alanine yielding pyruvate Alanine Dehydrogenase SAUSA300_1331 (*ald*)

(EC 1.4.1.1) SAUSA300_1655 (*ald2*)

Serine (1) Serine yielding pyruvate Serine Dehydratase SAUSA300_2469 (*sdaAA*)

(EC 4.3.1.7) SAUSA300_2470 (*sdaAB*)

Glycine (1) Glycine yielding Serine Serine Hydroxymethyl SAUSA300_2067

(via 5,10 methylenetetrahydrofolate Transferase

and glycine cleavage enzyme) (EC 2.1.2.1)

Glycine Dehydrogenase SAUSA300_1496

(EC 1.4.4.2) SAUSA300_1497

Glycine Cleavage System SAUSA300_0791 (*gcvH*)

H protein

Glycine Cleavage System SAUSA300_1498 (*gcvT*)

T protein

(EC 2.1.2.10)

Dihydrolipoamide Dehydrog- SAUSA300_0996 (*lpdA*)

enase

(EC 1.8.1.4)

(2) Serine yielding pyruvate Serine Dehydratase SAUSA300_2469 (*sdaAA*)

(EC 4.3.1.7) SAUSA300_2470 (*sdaAB*)

Threonine (1) Threonine yielding Glycine Threonine Aldolase SAUSA300_1214

(EC 4.1.2.5)

(2) Glycine yielding Serine Serine Hydroxymethyl SAUSA300_2067

(via 5,10 methylenetetrahydrofolate Transferase

and glycine cleavage enzyme) (EC 2.1.2.1)

Glycine Dehydrogenase SAUSA300_1496

(EC 1.4.4.2) SAUSA300_1497

Glycine Cleavage System SAUSA300_0791 (*gcvH*)

H protein

Glycine Cleavage System SAUSA300_1498 (*gcvT*)

T protein

(EC 2.1.2.10)

Dihydrolipoamide Dehydrog- SAUSA300_0996 (*lpdA*)

enase

(EC 1.8.1.4)

(3) Serine yielding pyruvate Serine Dehydratase SAUSA300_2469 (*sdaAA*)

(EC 4.3.1.7) SAUSA300_2470 (*sdaAB*)

Cysteine (1) Cysteine yielding Alanine Cysteine Desulferase SAUSA300_0820 (*sufS*)

(EC 2.8.1.7)

(2) Alanine yielding pyruvate Alanine Dehydrogenase SAUSA300_1331 (*ald*)

(EC 1.4.1.1)

**Amino acids yielding 2-oxoglutarate**

**Amino acid Reaction Enzyme FPR3757**

Glutamate (1) Glutamate yielding 2-oxoglutarate Glutamate Dehydrogenase SAUSA300_0861 (*gudB*)

(EC 1.4.1.3)

Glutamine (1) Glutamine yielding Glutamate Glutamate Synthase SAUSA300_0445 (*gltB*)

(EC 1.4.1.13) SAUSA300_0446 (*gltD*)

(2) Glutamate yielding 2-oxoglutarate Glutamate Dehydrogenase SAUSA300_0861 (*gudB*)

(EC 1.4.1.3)

Histidine (1) Histidine yielding Urocanate Histidine Ammonia Lyase SAUSA300_0008 (*hutH*)

(EC 4.3.1.3)

(2) Urocanate yielding 4-imidazolone- Urocanate Hydratase SAUSA300_2278 (*hutU*)

5-Propanoate (EC 4.2.1.49)

(3) 4-imidazolone-5-Propanoate yielding Imidazolone-5-Propanoate SAUSA300_2277 (*hutI*)

*N*-formimino-L-Glutamate hydrolase

(EC 3.5.2.7)

(4) *N*-formimino-L-Glutamate yielding Formiminoglutamate hydrolase SAUSA300_2281 (*hutG*)

Glutamate

(5) Glutamate yielding 2-oxoglutarate Glutamate Dehydrogenase SAUSA300_0861 (*gudB*)

(EC 1.4.1.3)

Arginine (1) Arginine yielding Ornithine Arginase SAUSA300_2114 (*rocF*)

(via arginase) (EC 3.5.3.1)

(2) Ornithine yielding Glutamate 5- Ornithine Aminotransferase SAUSA300_0187 (*rocD*)

Semialdehyde/Pyrroline 5- (EC 2.6.1.13)

Carboxylate

(3) Glutamate 5-Semialdehyde/ Pyrroline 5-Carboxylate SAUSA300_2491 (*rocA*)

Pyrroline 5-Carboxylate yielding dehydrogenase

Glutamate (EC 1.5.1.12)

(4) Glutamate yielding 2-oxoglutarate Glutamate Dehydrogenase SAUSA300_0861 (*gudB*)

(EC 1.4.1.3)

Arginine (1) Arginine yielding Citrulline Arginine Deiminase SAUSA300_2570 (*arcA1)*

(via arginine (EC 3.5.3.6) SAUSA300_0065 (*arcA2*)

deiminase)

(2) Citrulline yielding Ornithine Ornithine Carbamoyl- SAUSA300_2569 (*arcB1*)

transferase SAUSA300_0062 (*arcB2*)

(EC 2.1.3.3)

(3) Ornithine yielding Glutamate 5- Ornithine Aminotransferase SAUSA300_0187 (*rocD*)

Semialdehyde/Pyrroline 5- (EC 2.6.1.13)

Carboxylate

(4) Glutamate 5-Semialdehyde/ Pyrroline 5-Carboxylate SAUSA300_2491 (*rocA*)

Pyrroline 5-Carboxylate yielding dehydrogenase

Glutamate (EC 1.5.1.12)

(5) Glutamate yielding 2-oxoglutarate Glutamate Dehydrogenase SAUSA300_0861 (*gudB*)

(EC 1.4.1.3)

Proline (1) Proline yielding Pyrroline 5-Carbox- Proline Dehydrogenase SAUSA300_1711 (*putA*)

ylate/Glutamate 5-Semialdehyde (EC 1.5.99.8)

(2) Glutamate 5-Semialdehyde/ Pyrroline 5-Carboxylate SAUSA300_2491 (*rocA*)

Pyrroline 5-Carboxylate yielding dehydrogenase

Glutamate (EC 1.5.1.12)

(3) Glutamate yielding 2-oxoglutarate Glutamate Dehydrogenase SAUSA300_0861 (*gudB*)

(EC 1.4.1.3)

**Amino acids yielding oxaloacetate**

**Amino acid Reaction Enzyme FPR3757**

Aspartate (1) Aspartate yielding Oxaloacetate Aspartate Aminotransferase SAUSA300_1916 (*aspA*)

(EC 2.6.1.1)

Asparagine (1) Asparagine yielding Aspartate Asparaginase SAUSA300_1368 (*ansA*)

(EC 3.5.1.1)

(2) Aspartate yielding Oxaloacetate Aspartate Aminotransferase SAUSA300_1916 (*aspA*)

(EC 2.6.1.1)

**No catabolic pathway predicted**

Tryptophan

Isoleucine

Leucine

Lysine

Methionine

Phenylalanine

Tyrosine

Valine

^a^Catabolic pathways were predicted using BsubCyc and EcoCyc on the MetaCyc server (metacyc.org) and UniProt. *S. aureus* orthologues were detected using the USA300_FPR3757 genome sequence (CP000255).
