# Supplementary figures and images for: Amino Acid Catabolism in Staphylococcus aureus and the Function of Carbon Catabolite Repression
Source: mBio. 2017 Feb 14;8(1):e01434-16. doi: 10.1128/mBio.01434-16 (PMC5312079; doi:10.1128/mBio.01434-16)

Figure S1

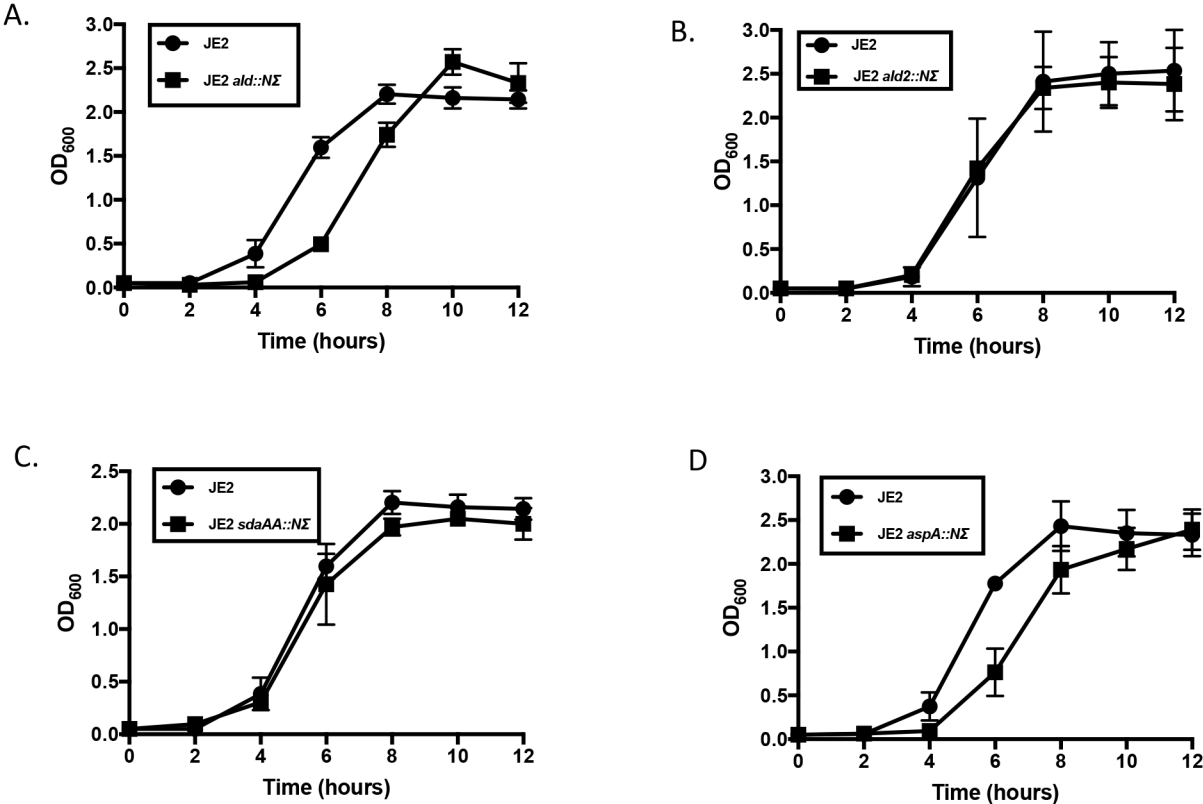

Figure S1

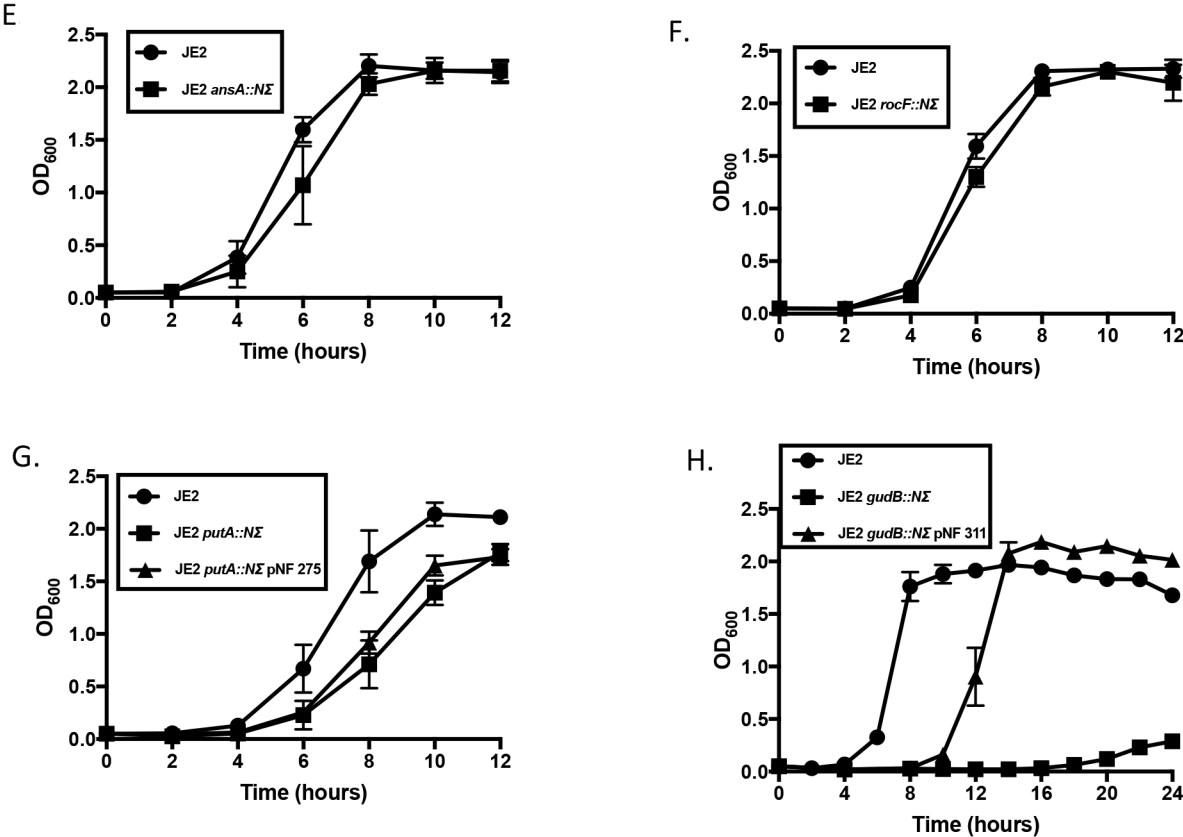

Figure S1

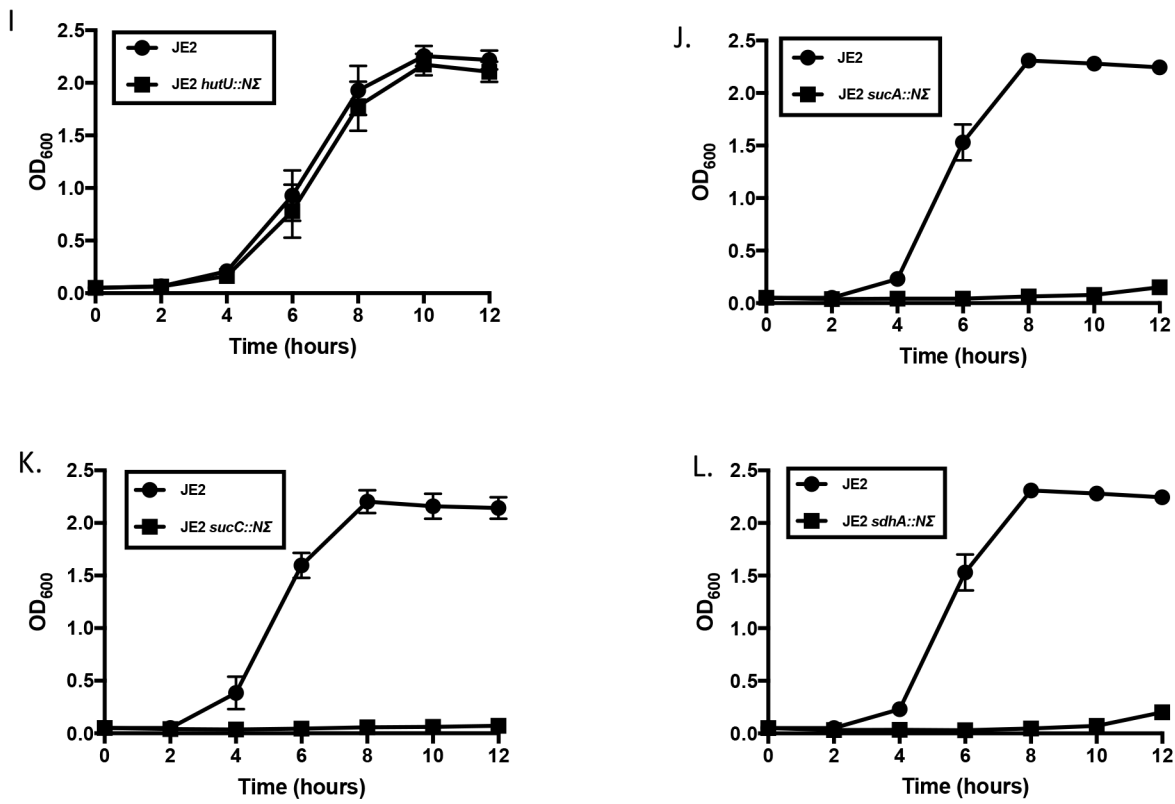

Figure S1

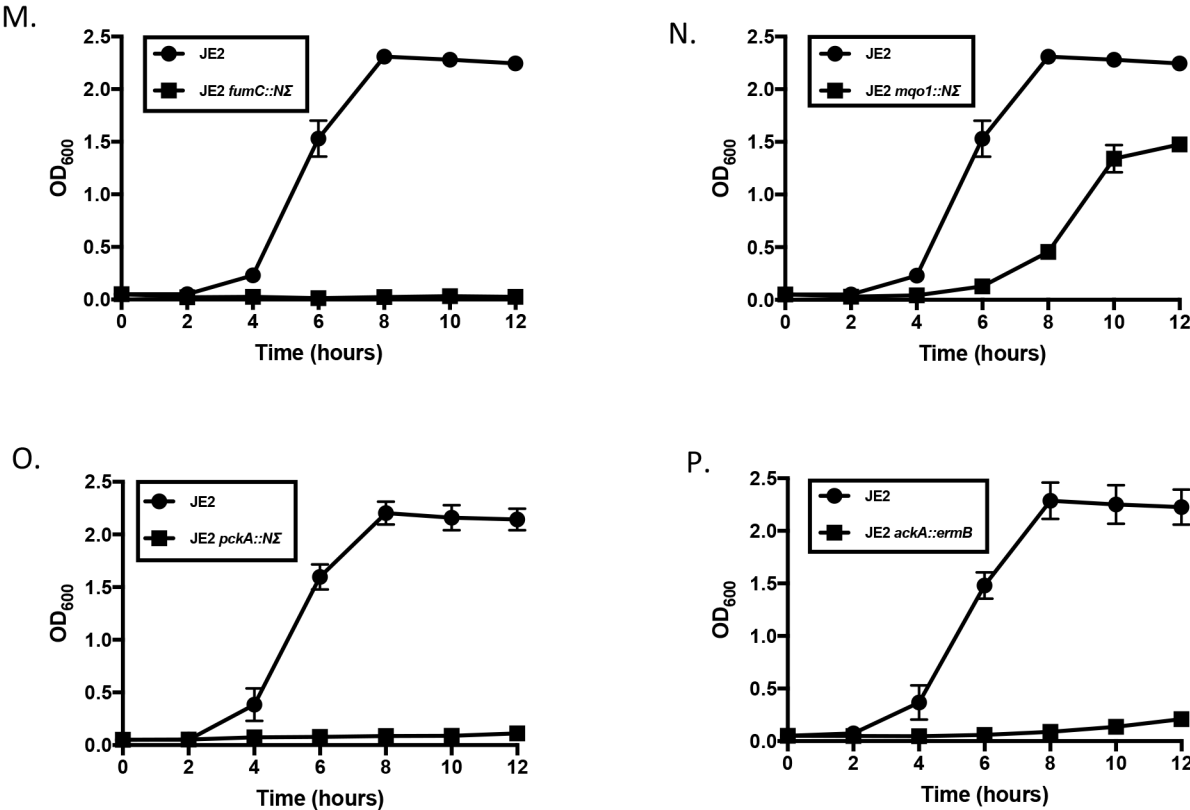

Figure S1

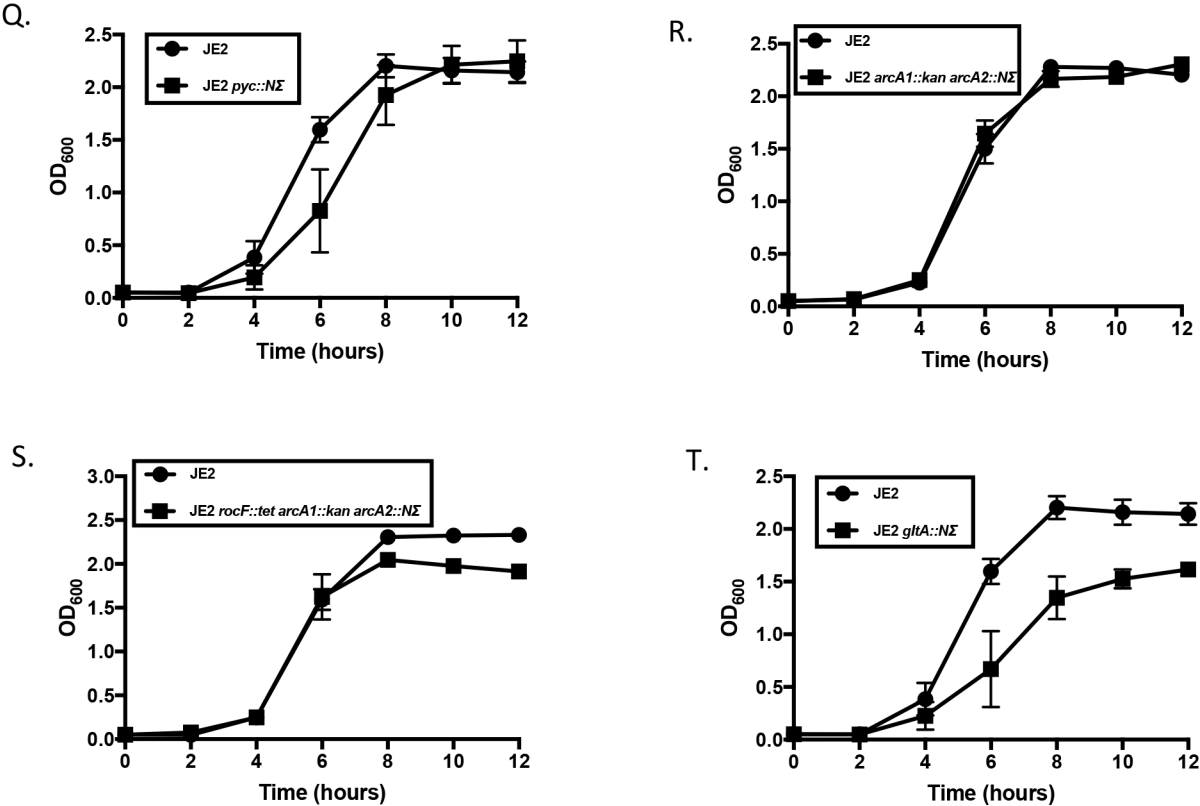

Figure S1

U.

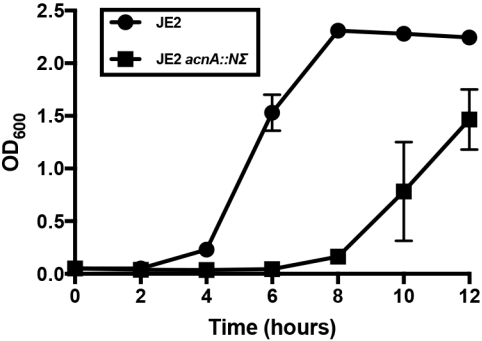

V.

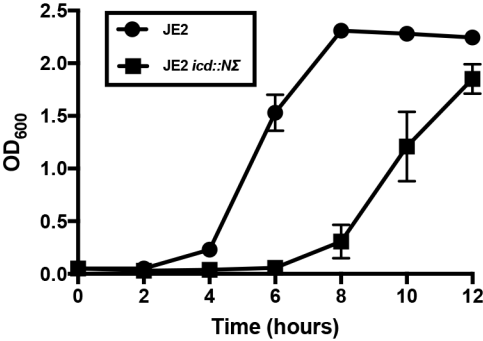

W.

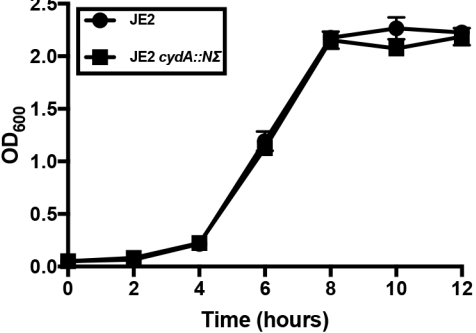

X.

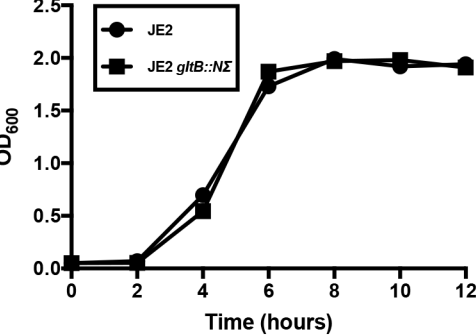

Figure S1

Y.

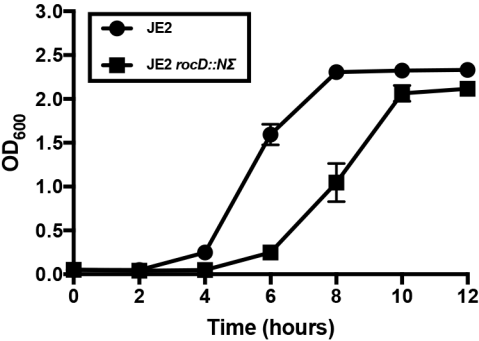

Z.

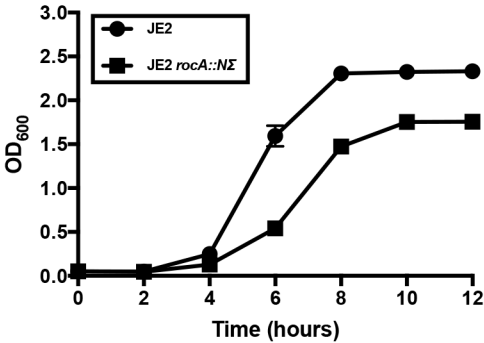

Supplement: FIG S1 [file mbo001173181sf1.pdf]

Figure S2

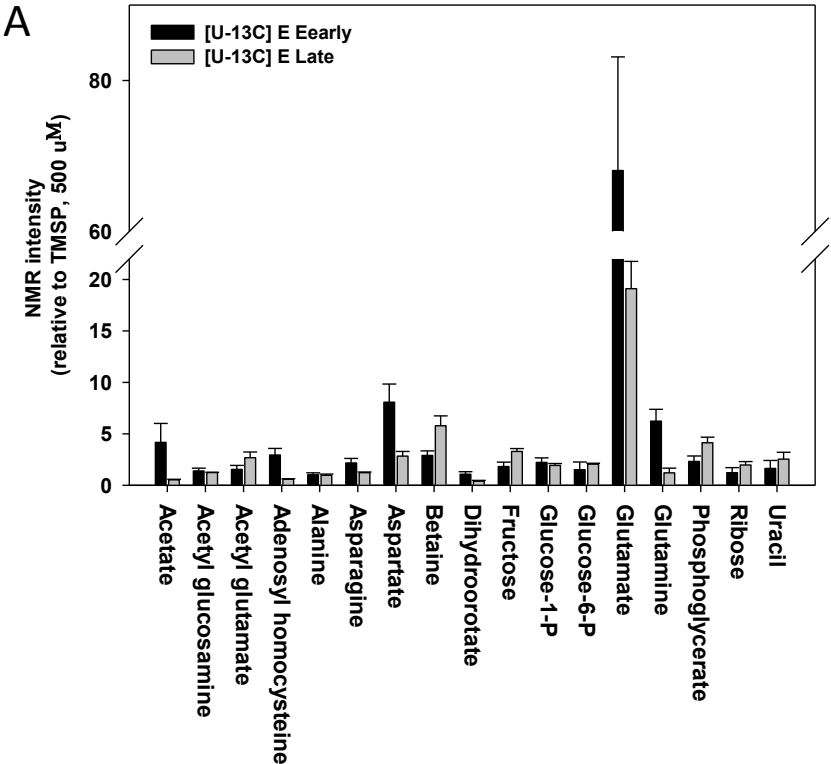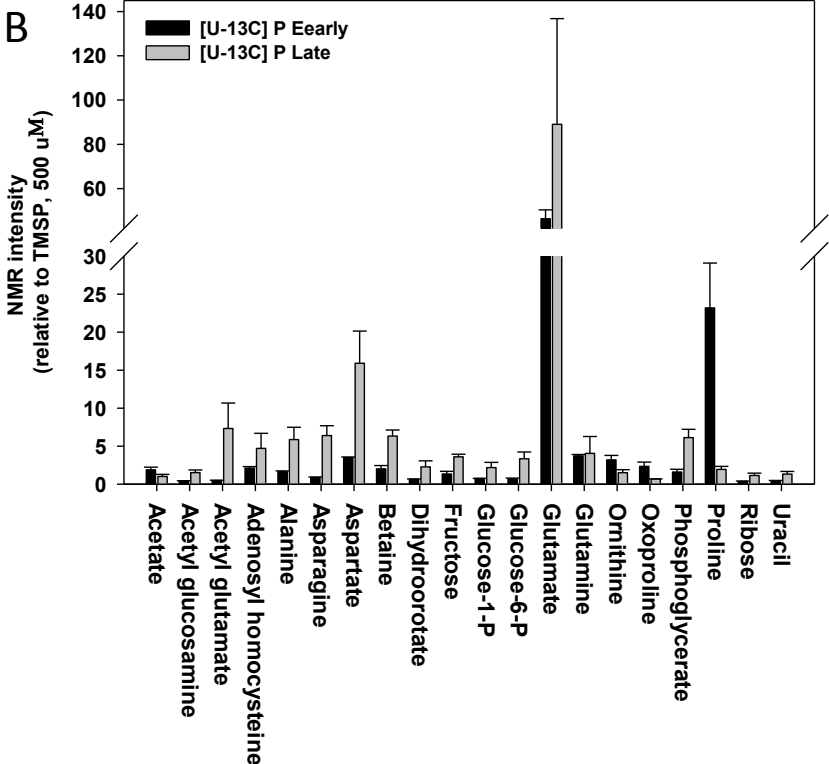

Figure S2

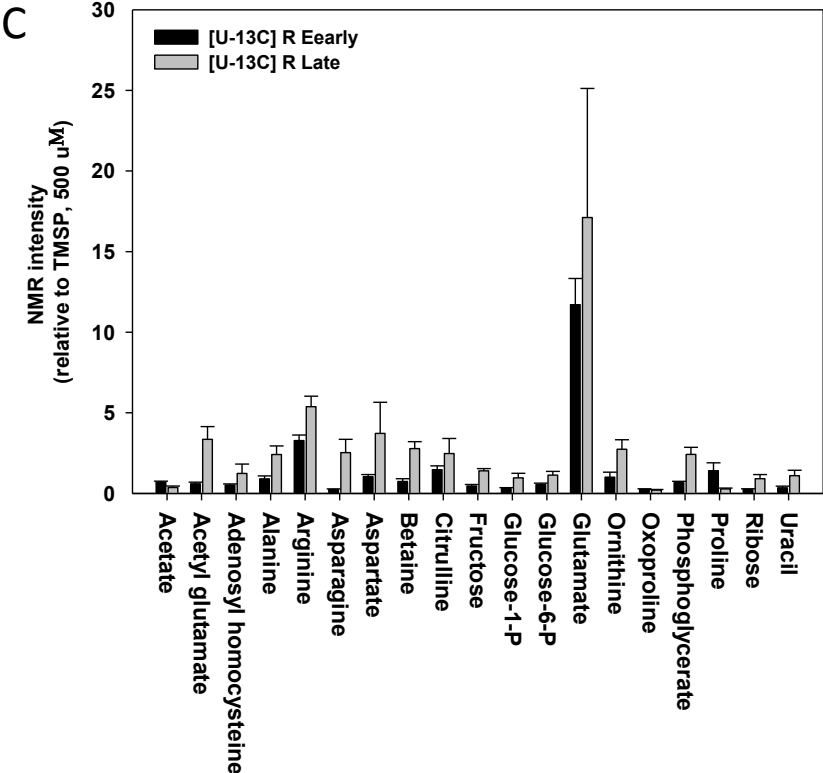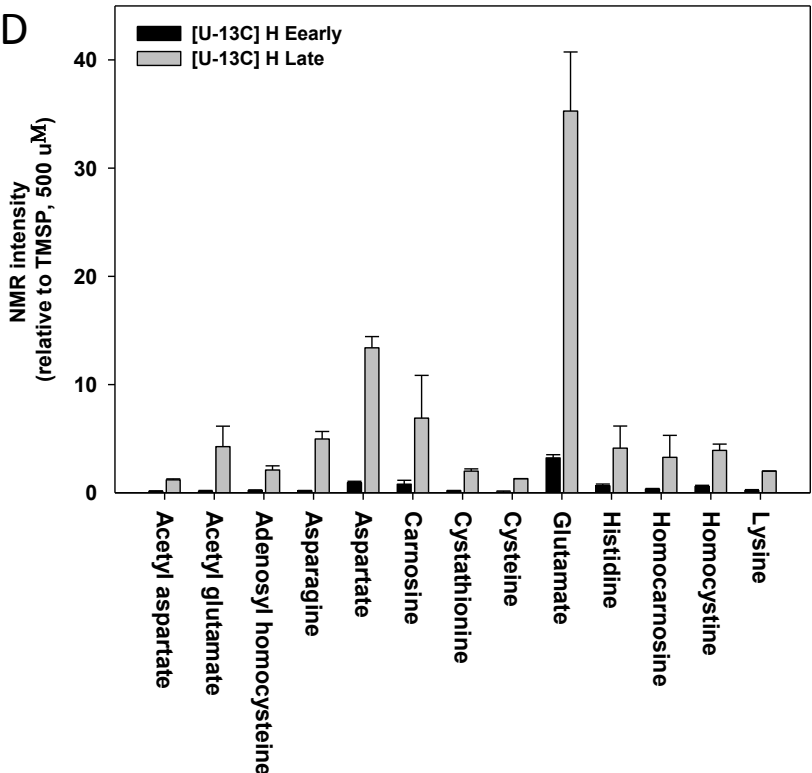

Figure S2

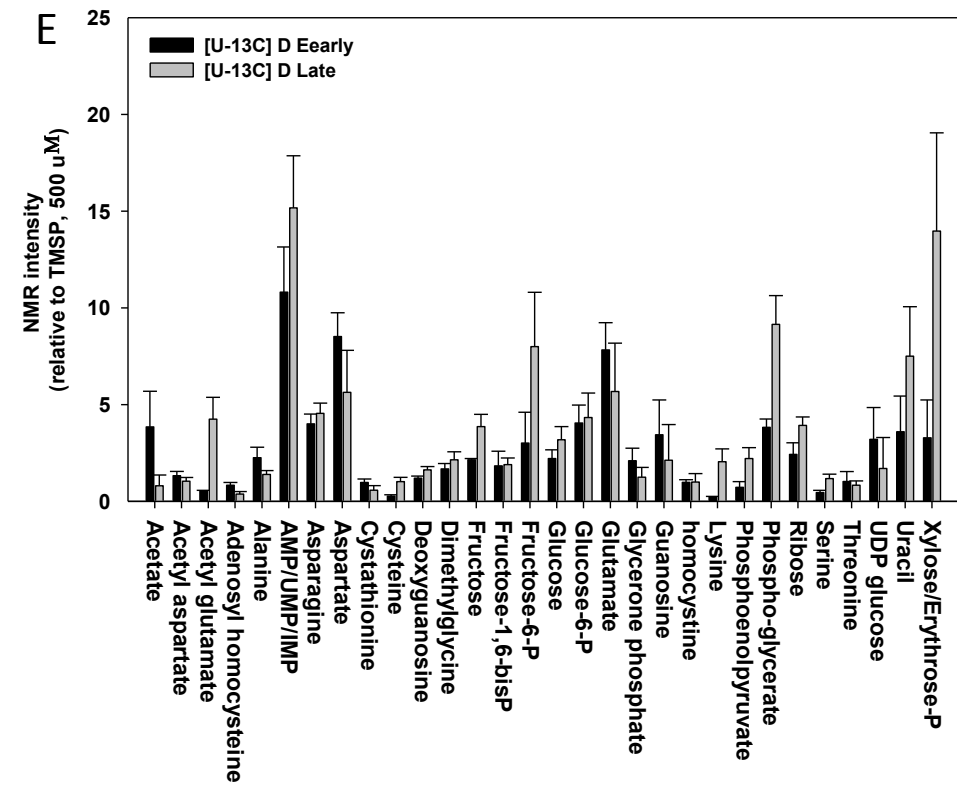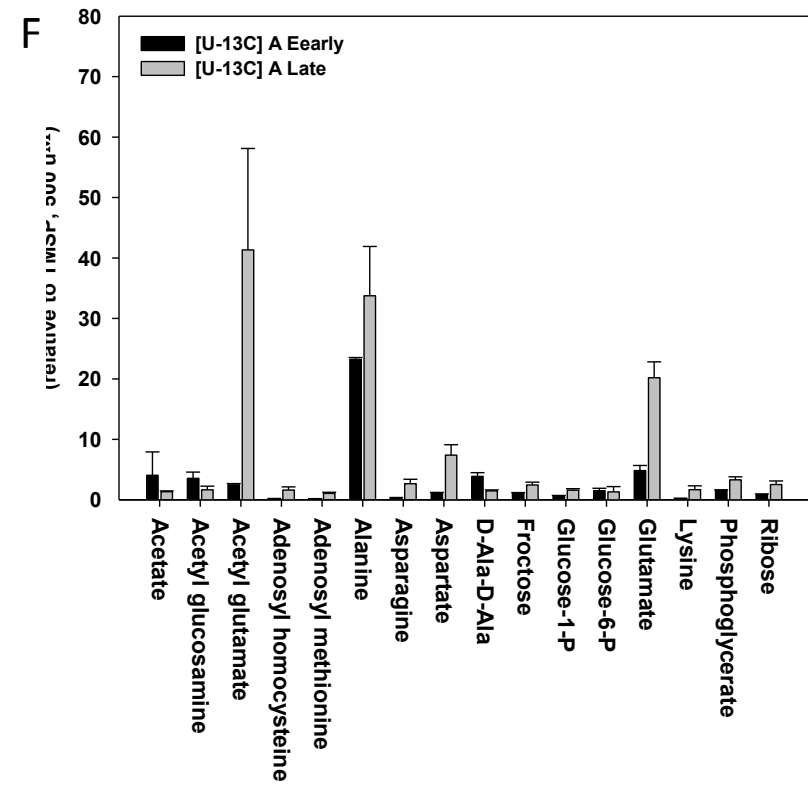

Figure S2

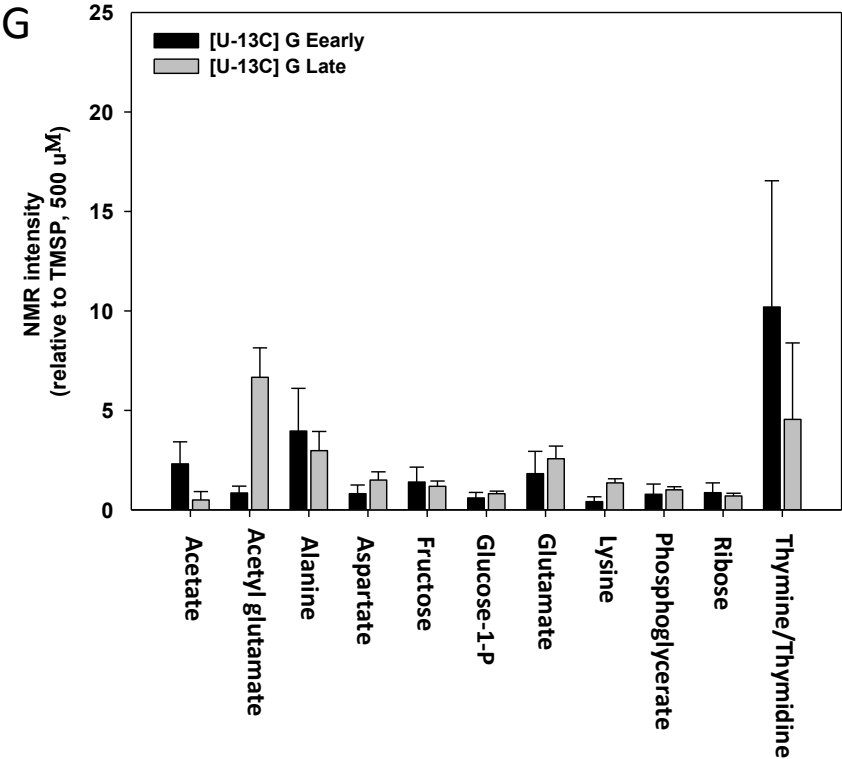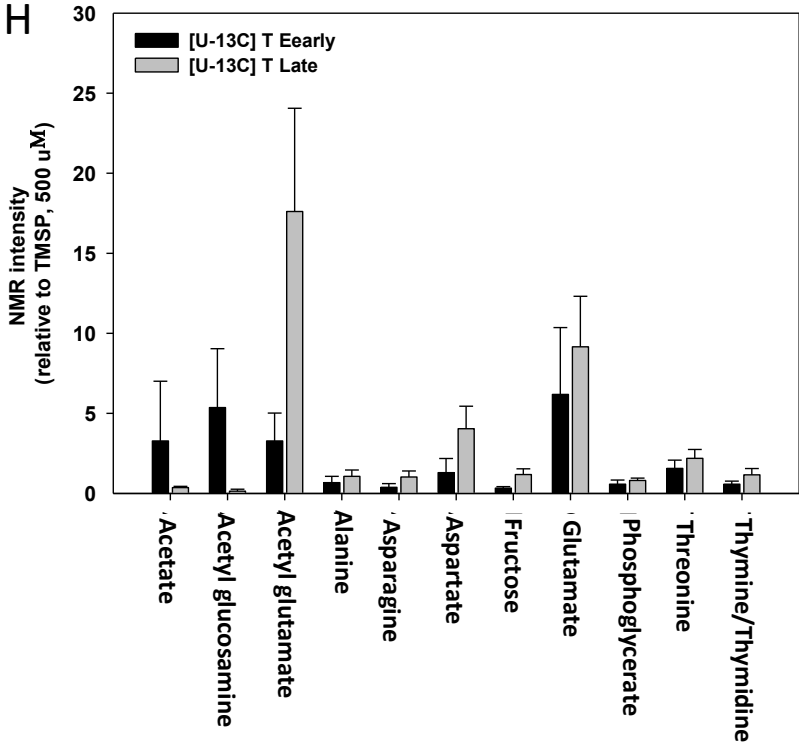

Figure S2

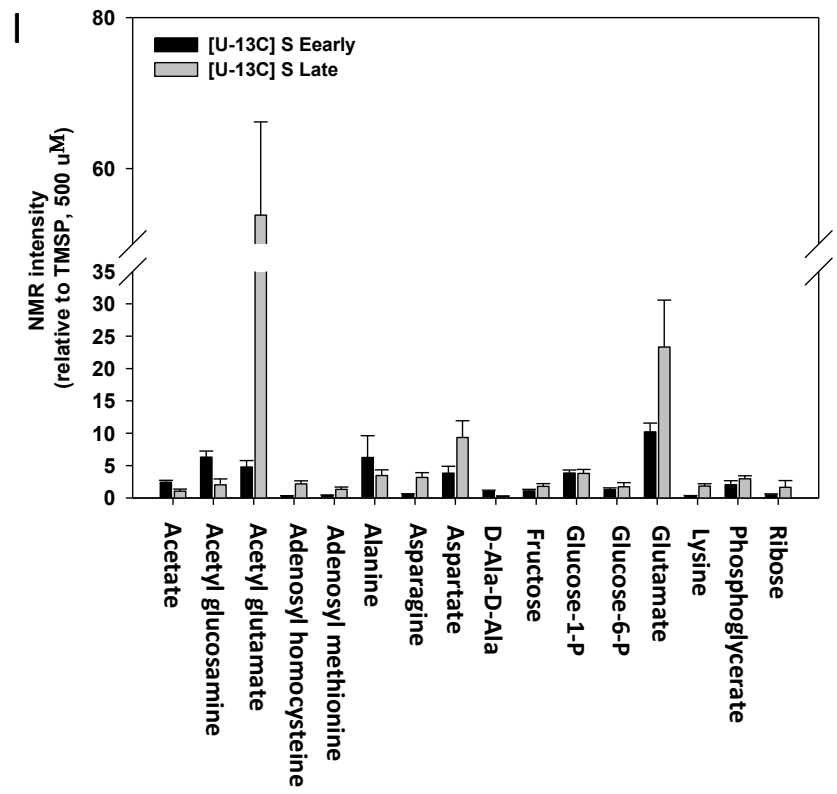

Supplement: FIG S2 [file mbo001173181sf2.pdf]

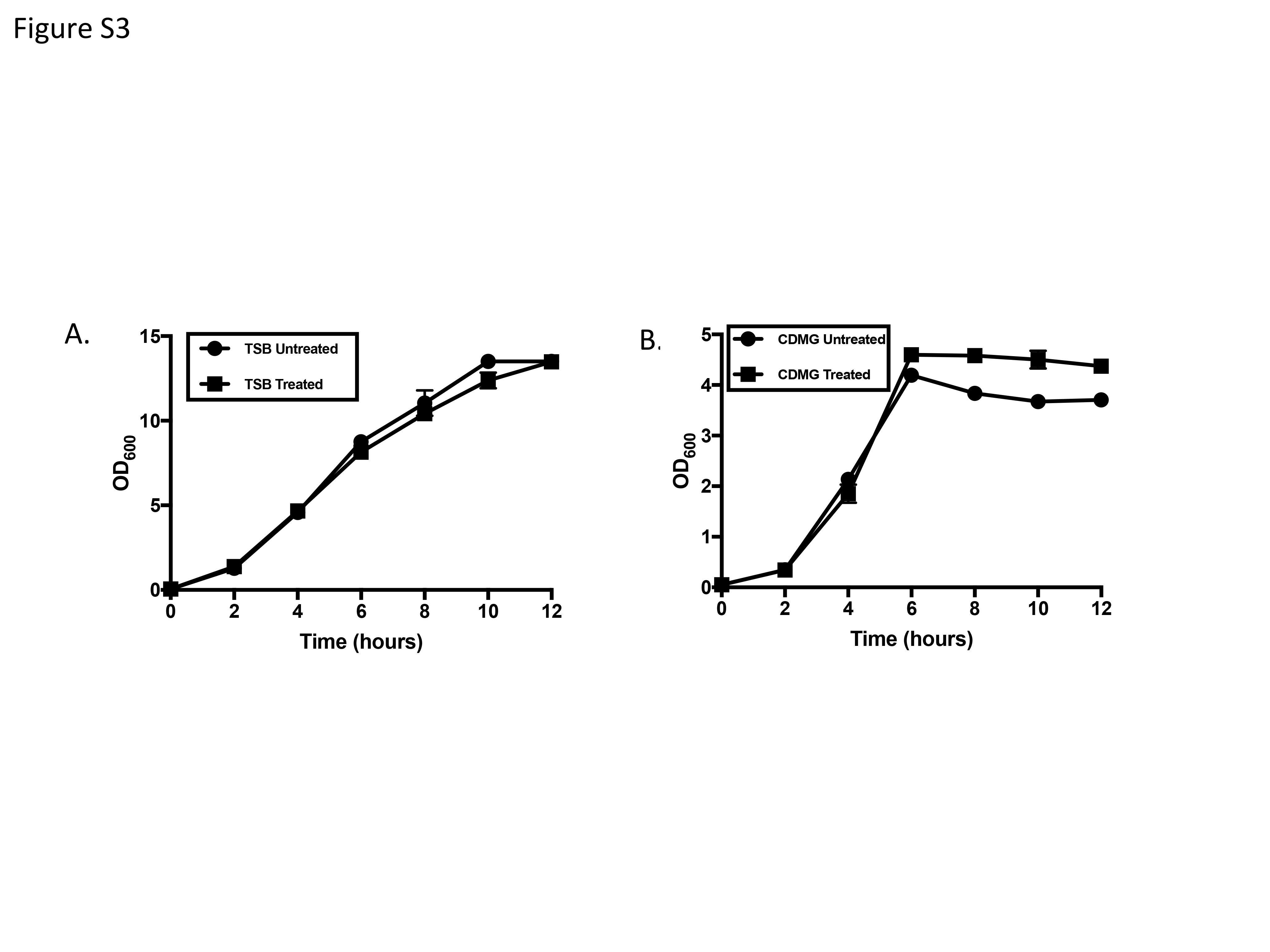

Supplement: FIG S3 [file mbo001173181sf3.tif]

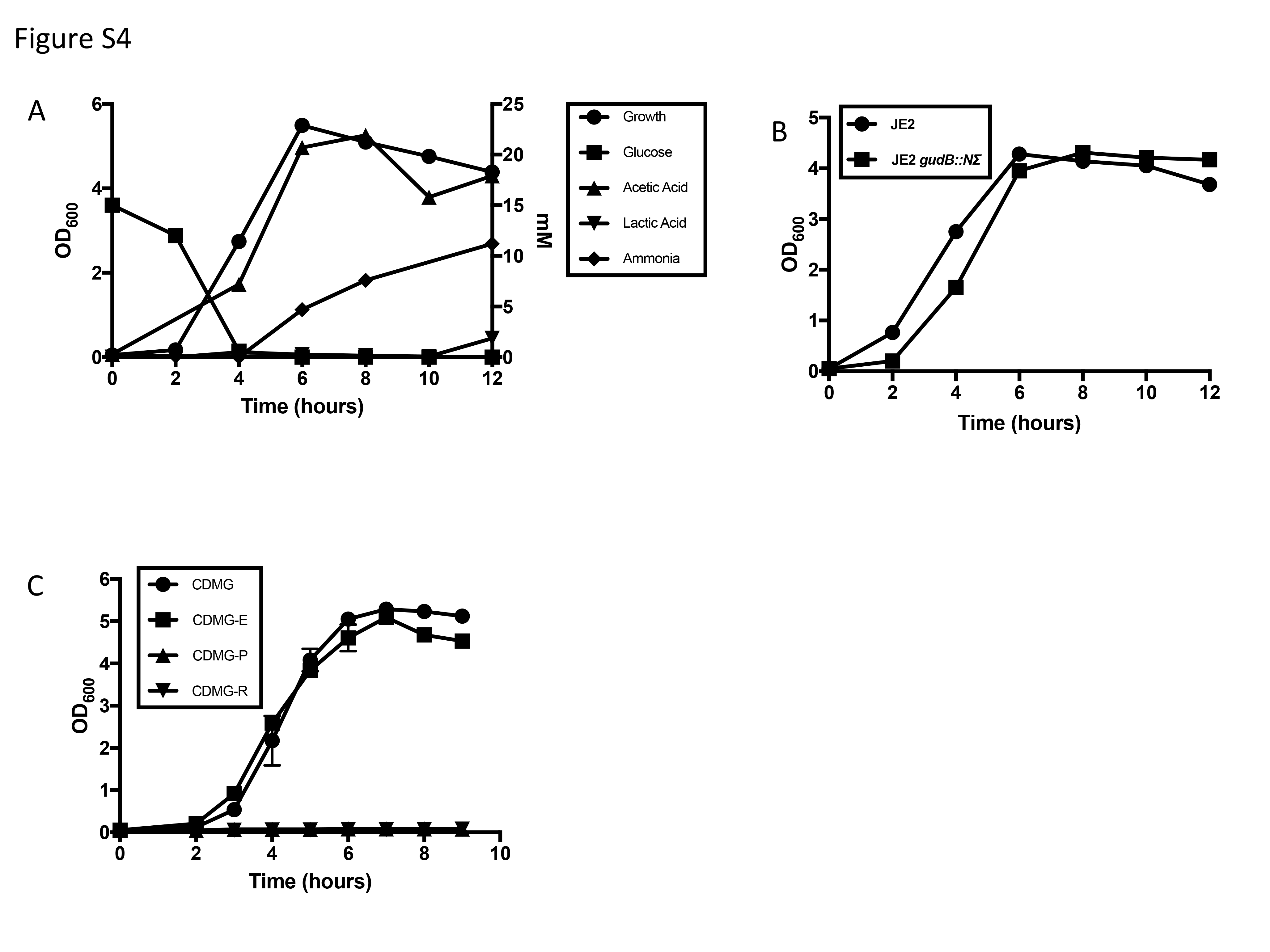

Supplement: FIG S4 [file mbo001173181sf4.tif]

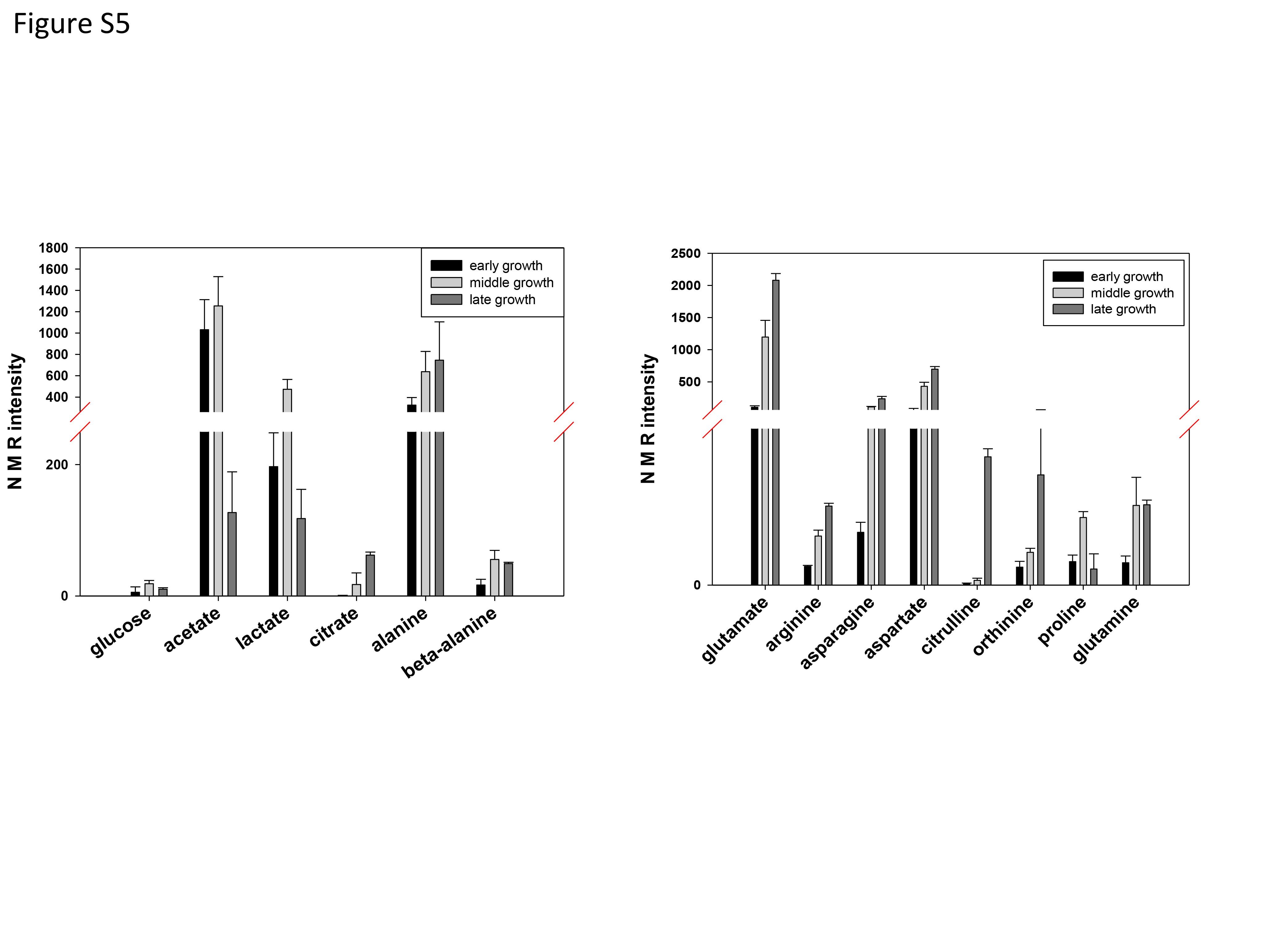

Supplement: FIG S5 [file mbo001173181sf5.tif]
