# Supplementary material for: Amino Acid Catabolism in Staphylococcus aureus and the Function of Carbon Catabolite Repression
Source: mBio. 2017 Feb 14;8(1):e01434-16. doi: 10.1128/mBio.01434-16 (PMC5312079; doi:10.1128/mBio.01434-16)
Supplement: TABLE S2 [file mbo001173181st2.docx]

| **Name** | **Sequence (5’ to 3’)** | **Target** |
| --- | --- | --- |
| 2838 | GCTTCATCAACATGGCGTAA | Forward primer to amplify *putA* |
| 2839 | ATGATGCGATGGTCATGTGT | Reverse primer to amplify *putA* |
| 2840 | TTCCACCCAGATGTTGATGA | Forward primer to amplify *gudB* |
| 2841 | GGCATTACCGAAACCTTGAA | Reverse primer to amplify *gudB* |
| 2932 | GAAGCAGTTGGTGATGCAGC | Forward primer to amplify *rocA* |
| 2933 | GCACGTGAACATGCTGAACA | Reverse primer to amplify *rocA* |
| 2034 | ATTGATGCAGCACAAGCAAG | Forward primer to amplify *rocD* |
| 2035 | CCGCTGTTAACCAAGGTCAT | Reverse primer to amplify *rocD* |
| 2836 | AGGTACCTGCTGTGAACATTGA | Forward primer to amplify *rocF* |
| 2837 | GAACGCCATCAACATTGCGT | Reverse primer to amplify *rocF* |
| 3100 | GGACAGATGACAGCCGGTAG | Forward primer to amplify *hutU* |
| 3101 | AGCCTGGGAAGTCAAAAGCA | Reverse primer to amplify *hutU* |
| 2344 | GGGCCC*CTGCAG*GCAATGGCACTATTAAAG | Forward *putA* contains PstI* restriction site |
| 2345 | GGGCCC*GGATCC*CATTGATTATTGTTCCAGACTCC | Reverse *putA* contains BamHI* restriction site |
| 2846 | GGCGGC*CTGCAG*ATGACTGAGAACAATAATTT | Forward *gudB* contains PstI* restriction site |
| 2848 | GGCGGC*GGATCC*TTATGCCCAACCACGATATGT | Reverse *gudB* contains BamHI* restriction site |

|  |  |
| --- | --- |
|  |  |

*Restriction site denoted in italics
